# Identification and Characterization of a Novel Insulin-like Receptor (*LvRTK2*) Involved in Regulating Growth and Glucose Metabolism of the Pacific White Shrimp *Litopenaeus vannamei*

**DOI:** 10.3390/biom14101300

**Published:** 2024-10-14

**Authors:** Zijian Liu, Jiawei Liu, Zijie Liu, Xiaowei Song, Su Liu, Fei Liu, Lin Song, Yi Gao

**Affiliations:** 1 College of Life Sciences, Qingdao Agricultural University, Qingdao 266109, China; 15764200096@163.com; 2 School of Marine Science and Engineering, Qingdao Agricultural University, Qingdao 266237, China; liujw1021@163.com (J.L.); 19862631038@163.com (Z.L.); 13406466771@163.com (X.S.); liufeizn@qau.edu.cn (F.L.); 3 Shandong Provincial Key Laboratory of Biochemical Engineering, College of Biological Engineering, Qingdao University of Science and Technology, Qingdao 266042, China; catmimidogdudu@163.com

**Keywords:** insulin receptor, RTK2, *Litopenaeus vannamei*, glucose metabolism, growth regulation, RNA interference

## Abstract

The insulin receptor (IR) plays a crucial role in the growth and metabolism of animals. However, there are still many questions regarding the IR in crustaceans, particularly their role in shrimp growth and glucose metabolism. In this study, we identified a novel insulin-like receptor gene in *Litopenaeus vannamei* and cloned its full length of 6439 bp. This gene exhibited a highly conserved sequence and structural characteristics. Phylogenetic analysis confirmed it as an unreported RTK2-type IR, namely, *LvRTK2*. Expression pattern analysis showed that *LvRTK2* is primarily expressed in female reproductive and digestive organs. Through a series of in vivo and in vitro experiments, including glucose treatment, exogenous insulin treatment, and starvation treatment, *LvRTK2* was confirmed to be involved in the endogenous glucose metabolic pathway of shrimp under different glucose variations. Moreover, long-term and short-term interference experiments with *LvRTK2* revealed that the interference significantly reduced the shrimp growth rate and serum glucose clearance rate. Further studies indicated that *LvRTK2* may regulate shrimp growth by modulating the downstream PI3K/AKT signaling pathway and a series of glucose metabolism events, such as glycolysis, gluconeogenesis, glycogen synthesis, and glycogenolysis. This report on the characteristics and functions of *LvRTK2* confirms the important role of RTK2-type IRs in regulating shrimp growth and glucose metabolism.

## 1. Introduction

Insulin is one of the most well-known hormones and is the key hormone that regulates blood glucose stability [1,2]. Since its discovery, the molecular mechanisms of insulin action have been the focus of research [3]. Insulin exerts its effects by binding to insulin receptors (IRs) on the surface of target cells, initiating the insulin signaling pathway [4]. Subsequently, the insulin signaling pathway regulates the uptake of glucose, fatty acids, and amino acids into the liver, muscle, and adipose tissue, storing nutrients in the form of glycogen, lipids, and proteins [5]. All these processes are triggered by insulin activating IRs [6,7].

The IR is a transmembrane protein belonging to the superfamily of tyrosine kinase receptors. It exists as covalently bound receptor dimers α2β2 at the cell surface [8]. Insulin can bind with the ectodomain of IRs to trigger a signal transduction cascade [6,7], primarily through the phosphoinositide 3-kinase (PI3K)/protein kinase B (Akt/PKB) pathway [9]. Generally, insulin-related peptides act on two different types of IR: receptor tyrosine kinase (RTK) and leucine-rich repeat G protein-coupled receptor (LGR) [10].

The role of IRs has been extensively studied in vertebrates, primarily involving the regulation of metabolic activity, cell proliferation, and growth [11,12,13]. In humans, IRs have been found to play a key role in multiple chronic diseases, including obesity, Type 2 diabetes mellitus (T2DM), neurodegenerative disorders, Alzheimer’s disease (AD), and cancers [11,14,15,16]. In invertebrates, the role of insulin-like receptors (IR homolog in invertebrates) and the insulin pathway is more diverse, encompassing not only the regulation of glucose homeostasis but also development, reproduction, lifespan, wing polymorphism, morphological diversity, circadian rhythmicity, and behavior [4,8,17,18,19].

However, research on IRs in crustaceans is very limited. In fact, it was not until 2020 that the RTK-type IRs of decapoda crustaceans were classified into four categories, RTK1-4 [20]. These four types of RTKs have been shown to belong to different subfamilies and are considered to be four distinct homologs that evolved independently from the RTKs found in insects [20]. Prior to this, research on crustacean IRs generally referred to the study subjects as “insulin-like receptors”, but, in reality, most studies focused on only one type of IR, which is RTK4 [21,22,23,24]. RTK4 is considered to be the receptor for insulin-like androgenic gland hormone (IAG), which was the first identified insulin-like peptide in crustaceans, identified by Manor et al. in 2007 [25]. IAG is believed to play a crucial role in sexual differentiation [26]. RTK4 is predominantly expressed in the male reproductive organs, and its role in sexual differentiation has been studied in species such as *Fenneropenaeus chinensis* [21], *Sagmariasus verreauxi* [22], *Litopenaeus vannamei* [23], and *Scylla paramamosain* [24]. However, this also means that our understanding of the functions of the other three types of RTK (RTK1-3) is very limited at present.

Through sequence alignment, it was found that only two studies have analyzed non-RTK4 in crustaceans [27,28]. These include the study by Sharabi et al. in 2016, where they identified and characterized a gene encoding IR (actually RTK1) in *Macrobrachium rosenbergii* called Mr-IR. The study found that silencing *MrIR* resulted in enlargement of the androgenic gland (AG) in males and increased production of IAG, but no sex reversal occurred [27]. In 2022, Li et al. identified an IR (actually RTK1) in *Eriocheir sinensis* and found an association between *EsIR* and limb regeneration through the injection of an IR inhibitor [28]. Besides these two studies on RTK1, there are currently no relevant studies involving the other two RTK classes (RTK2 and RTK3). Furthermore, there have been no reports on the involvement of all RTKs in the growth and carbohydrate metabolism processes of crustaceans. Research on insects has shown functional differences between two copies of IRs in insects [29,30]. For instance, different expression patterns and functions of the two IRs were observed during the development and reproduction of *Tribolium castaneum* [7]. In the wing development of planthoppers, two IRs were found to have antagonistic effects [31]. In *Apis mellifera*, *Bombus terrestris*, and *Solenopsis invicta*, the two copies of IRs were found to have caste- and tissue-specific expressions [32,33]. These findings have sparked our curiosity about the functions of the four classes of RTKs in crustaceans and whether there are functional differences among them. It is noteworthy that, unlike studies on insects that typically focus on pest control [15], research on IRs in decapod crustaceans is closely related to promoting their growth, as these animals usually have significant economic value [34]. Therefore, it is necessary to investigate the role of RTK in the growth and carbohydrate metabolism of crustaceans.

The Pacific white shrimp (*Litopenaeus vannamei*) is the most economically important shrimp species globally, accounting for over 50% of total shrimp production (FAO, 2024) [35]. Consequently, research on its growth mechanism has attracted significant attention [36]. As an upstream factor in the insulin signaling pathway, the IR is considered one of the key factors associated with shrimp growth [34]. However, despite the completion of the sequencing of the *L. vannamei* genome [36], the sequences of most IRs in *L. vannamei* have not been publicly available.

Therefore, we designed this study to identify the IR gene, establish the relationship between the IR and shrimp growth, and explore the mechanisms by which IR affects growth. As a result, we discovered a previously unreported novel IR/insulin-like receptor gene in *L. vannamei*, which was identified as the second type of RTK (RTK2). Subsequently, we conducted in vivo and in vitro experiments, including glucose treatment, exogenous insulin treatment, and starvation treatment, to investigate the role of *LvRTK2* in *L. vannamei* glucose metabolism. Additionally, long-term and short-term *LvRTK2* RNA-interference (RNAi) experiments provided insights into the association between this gene and shrimp growth, as well as the downstream pathways and molecular events. This research is of significant importance in advancing our understanding of the insulin signaling pathway and growth mechanisms in decapod crustaceans.

## 2. Materials and Methods

### 2.1. Experimental Animals

Healthy *L. vannamei* were cultured in seawater aquaculture pools at the College of Marine Science and Engineering, Qingdao Agricultural University, and were acclimated for more than two weeks prior to this study. The water temperature in the culture system was maintained at a stable 23 °C, with feed provided three times a day. Fresh seawater was replaced daily, and sufficient oxygen levels were ensured. All animal care and handling procedures were approved by the Animal Care Committee of Qingdao Agricultural University (approval No. 2018-192).

### 2.2. Amplification, Cloning, and Sequencing of Insulin-like Receptor (IR) Gene in L. vannamei

Total RNA was extracted from different tissues of *L. vannamei* (including eyestalk, epidermis, gill, heart, stomach, hepatopancreas, gut, ovary, oviduct, testis, vas deferens, sperm atophore, thoracic ganglia, ventral nerve cord, muscle, and hemocyte) using the RNAiso Plus reagent (TaKaRa, Tokyo, Japan). Each tissue was pooled from the corresponding tissues of six shrimp with three biological replicates (n = 18). The yield and purity of each RNA sample with 600–1000 ng/μL concentrations were determined using a NanoDrop™ 2000 spectrophotometer (Thermo Fisher Scientific, Waltham, MA, USA). The integrity of RNA samples was assessed using an Agilent 2100 Bioanalyzer (Agilent Technologies, Palo Alto, CA, USA) and verified by RNase-free agarose gel electrophoresis. Additionally, DNase (TaKaRa, Tokyo, Japan) was applied to remove DNA contamination from the RNA. Subsequently, RNA was reverse transcribed into cDNA using the PrimeScript™ RT reagent kit (TaKaRa, Tokyo, Japan), and the cDNA was used as a template to attempt amplification of the full-length sequence of *LvIR*. The PCR reaction process for the full-length amplification is as follows: pre-denaturation at 95 °C for 3 min; denaturation at 95 °C for 30 s, annealing at 48 °C for 30 s, and extension at 72 °C for 2.5 min, for a total of 34 cycles; followed by an extension at 72 °C for 5 min. Primers for *LvIR* were designed based on the genomic and transcriptomic data of *L. vannamei*. (NCBI accession number: GCF_003789085.1, SRP094135, SRP061180, and unpublished laboratory data). Ultimately, the full-length sequence of *LvIR* was obtained from the tissue of the ventral nerve cord; the primer information used is listed in Table 1. The PCR products were purified via gel extraction and then subjected to TA cloning. The cloned products were sequenced by Sangon Biotech (Shanghai, China) Co., Ltd.

### 2.3. Sequence Characterization of LvRTK2

The open reading frame (ORF) sequence of *LvRTK2* was predicted using the ORFfinder (https://www.ncbi.nlm.nih.gov/orffinder/, accessed on 28 August 2023). The amino acid sequence of *LvRTK2* was predicted using ExPASy (https://www.expasy.org, accessed on 1 September 2023). The functional domains of the obtained sequence were analyzed using SMART (http://smart.embl.de/, accessed on 10 September 2023) and Pfam (https://pfam.xfam.org/, accessed on 10 September 2023). The transmembrane regions were confirmed using the TMHMM Server (www.cbs.dtu.dk/services/TMHMM/, accessed on 25 September 2023). The three-dimensional structure of the extracellular region of *LvRTK2* was predicted via homology modeling using the online tool Swiss-model (https://swissmodel.expasy.org/, accessed on 12 October 2023). The sequenced ORF was mapped to the genomic data of *L. vannamei* using the BLAST+ v2.14.0 program to identify exons and introns. The amino acid sequences of IR homologs from other decapoda species were downloaded from the NCBI database. A multiple sequence alignment and the Neighbor-Joining (NJ) phylogenetic tree with 1000 bootstrap replicates were performed using MEGA 6.0. The active site of the tyrosine kinases was predicted using the online database Prosite (https://prosite.expasy.org/, accessed on 1 September 2023). The glycosylation and phosphorylation sites in *LvRTK2* were predicted using the NetNGlyc Server (www.cbs.dtu.dk/services/NetNGlyc/, accessed on 9 December 2023) and the NetPhos Server (www.cbs.dtu.dk/services/NetPhos/, accessed on 9 December 2023), respectively.

### 2.4. Expression Analysis of LvRTK2 in Different Tissues, Early Development Stages, and Molting Stages

The relative expression levels of *LvRTK2* in sixteen adult tissues of *L. vannamei*, including eyestalk, epidermis, gill, heart, stomach, hepatopancreas, gut, ovary, oviduct, testis, vas deferens, sperm atophore, thoracic ganglia, ventral nerve cord, muscle, and hemocyte, were examined by quantitative real-time PCR (qRT-PCR). Each tissue was pooled from the corresponding tissues of six shrimp with three biological replicates (n = 18). qRT-PCR was performed using the TB Green^®^ Premix Ex Taq™ II (TaKaRa, Tokyo, Japan) on a QuanStudio™ 5 Real-Time PCR Instrument (Thermo Fisher Scientific, Waltham, MA, USA). The 18S rRNA was used as an internal control. The steps for the qRT-PCR reaction are as follows: first, pre-denaturation is performed at 95 °C for 30 s. Then, a total of 40 cycles are carried out, including denaturation at 95 °C for 15 s, annealing for 30 s (the annealing temperature is set according to the primer’s annealing temperature shown in Table 1), and extension at 72 °C for 30 s. The primer information for qRT-PCR is listed in Table 1. Each sample was run in triplicate on the qRT-PCR system, and the relative expression levels were calculated using the 2^−ΔΔCt^ method [37].

The relative expression levels of *LvRTK2* at nine early developmental stages and eight molting stages are derived from newly assembled transcriptomic data. The raw data for the early developmental stages and molting periods of *L. vannamei* come from the study by Gao et al. (2017) [38], with accession numbers in the NCBI SRA database: SRP094135 for the nine early developmental stages and SRP061180 for the eight molting stages. First, the quality of the raw reads was checked using FastQC v0.11.5 [39], and low-quality data and adapters were filtered out using the Bowtie2 v2.2.8 program [40]. Then, the data were aligned to the *L. vannamei* genome using the HISAT2 v2.0.5 software package with default parameters [41]. The reference genome was based on the latest version of *L. vannamei* genome from NCBI (GCF_003789085.1), with some modifications made, including the addition of the newly acquired *LvRTK2* sequences (PP932464.1) and the replacement of four segments (XM_027362264.1, XM_027370182.1, XM_027362239.1, and XM_027362303.1). Subsequently, StringTie v1.3.1 was used to calculate the fragments per kilobase of transcript per million mapped reads (FPKM) values for each transcript region [42].

### 2.5. In Vivo Glucose Injection Experiment in Shrimp

To detect the changes in glucose levels in the serum of shrimp and the expression, after the injection, of exogenous glucose, we selected a total of 432 healthy shrimp with an average weight of 20.45 ± 2.58 g for the glucose injection experiment. After a 12 h fasting period, a glucose solution at a dose of 1.0 g/kg body weight was injected into the shrimp using a microsyringe. Blood samples were collected at 5, 15, 30, 60, 180, and 360 min after injection using an optimized sugar-free anticoagulant to eliminate the impact of glucose. The control group was injected with PBS instead of the glucose solution, while other conditions remained unchanged. At each time point, blood samples were collected from 12 shrimp per group and mixed with twice the volume of anticoagulant. Each group of samples was collected in three biological replicates. After blood collection, the shrimp’s hepatopancreas, stomach, and muscle tissues were quickly removed from the shrimp and rapidly frozen in liquid nitrogen. Blood samples were centrifuged (1000 rpm, 4 °C, 5 min), and the upper serum was analyzed for glucose concentration using the glucose detection kit (F006-1-1) purchased from Jiancheng Bioengineering Institute (Nanjing, China). The deposited blood cells, along with hepatopancreas, stomach, and muscle tissues, were collected for total RNA extraction, which was then reverse transcribed into cDNA for qRT-PCR detection of *LvRTK2* gene expression. The hepatopancreas and stomach are classic digestive tissues, the hemocyte is the direct effector of blood glucose regulation, and muscle is the primary tissue responsible for the weight gain in shrimp. Therefore, we selected these four types of tissues for measurement.

### 2.6. In Vitro Glucose Addition Experiment on Primary Hepatopancreas Cells

The primary hepatopancreatic cell culture of *L. vannamei* follows the method described by Chen et al. (2015) and Duan et al. (2018) [43]. In brief, the primary cell cultures were maintained at 25 °C using an L15 medium supplemented with glutamine (Biosharp, Beijing, China). After 24 h of culture to stabilize, the control group and experimental group were treated with 50 μL PBS solution and 50 μL glucose solution (200 g/L), respectively. After 60 min of treatment, the cells were collected to extract total RNA, which was then reverse-transcribed into cDNA. The expression level of *LvRTK2* was measured using qRT-PCR. All cell experiments included three biological replicates and three technical replicates, and the results are presented as mean ± standard deviation (SD).

### 2.7. In Vivo Exogenous Insulin Injection Experiment in Shrimp

A total of 360 healthy shrimp with an average weight of 10.93 ± 2.08 g were selected for the exogenous insulin injection experiment. All shrimp were randomly divided into two groups and fasted for 12 h to eliminate initial blood glucose differences caused by diet. Subsequently, a glucose solution of 1.0 g/kg body weight was then injected individually. After 10 min, the experimental group received an injection of 8.0 IU/kg of bovine insulin (G-clone, Beijing, China), while the control group was injected with an equivalent volume of PBS. Blood and tissue samples (hepatopancreas, stomach, and muscle) were collected from both the experimental and control groups at 15, 30, 60, 180, and 360 min post-injection. Sampling collection, glucose measurement, and gene expression analysis followed the same procedure outlined in Section 2.5.

### 2.8. In Vitro Exogenous Insulin Addition Experiment on Primary Hepatopancreas Cells

After culturing the primary hepatopancreatic cells for 24 h, 50 μL of glucose solution (200 g/L) was added to each well. After 10 min, the control group was added with 50 μL of PBS, while the experimental group received 50 μL of bovine insulin (1.6 IU/mL). After 60 min of treatment, total RNA was extracted from the cells, followed by reverse transcription to obtain cDNA, and the relative expression of the *LvRTK2* was measured.

### 2.9. In Vivo Starvation Treatment Experiment in Shrimp

A total of 360 healthy shrimp with an average weight of 10.28 ± 1.93 g were selected for the starvation treatment experiment. All shrimps were randomly divided into two groups: the control group was fed normally, while the experimental group was not fed. Blood and tissue samples (hepatopancreas, stomach, and muscle) were extracted from the experimental group and control group at 16, 32, 64, 128, and 256 h after the starvation treatment. Sampling collection, glucose measurement, and gene expression analysis followed the same procedure outlined in Section 2.5.

### 2.10. In Vitro Starvation Simulation Experiment on Primary Hepatopancreas Cells

The isolated primary hepatopancreatic cells were divided into a control group and an experimental group. The experimental group was cultured in PBS solution (50 μL per well) to simulate a starvation environment, while the control group received an additional 50 μL of glucose solution (200 g/L) in the PBS. After 24 h of incubation, the cells were collected to extract total RNA, reverse transcribed into cDNA, and the relative expression level of *LvRTK2* was determined.

### 2.11. Effect of Long-Term RNA Interference of LvRTK2 on Shrimp Growth

The preparation of double-stranded RNA (dsRNA) followed the procedures described by Guo et al. (2019) [44,45], using the TranscriptAid T7 High Yield Transcription Kit (Thermo Fisher Scientific, Waltham, MA, USA). The primers used in the experiment are listed in Table 1. The optimal interference concentration of dsLvRTK2 was determined through a preliminary experiment. A total of 180 healthy shrimp (average weight of 1.20 ± 0.49 g) were equally divided into three groups (PBS group, dsEGFP group, and dsLvRTK2 group) and injected with 10 μL of PBS, 10 μL of dsEGFP (4 µg/10 µL), and 10 μL of dsLvRTK2 (4 µg/10 µL), respectively. The injection of shrimp was performed every four days, with a total duration of 20 days. On the 12th, 16th, and 20th days after injection, the average weight of the shrimp in the three groups was measured, and the results are presented as mean ± SD. At the end of the experiment, the hepatopancreas tissues of the control group (PBS group) and the dsLvRTK2 group were dissected and preserved in liquid nitrogen for subsequent experiments.

### 2.12. Effect of Short-Term RNA Interference of LvRTK2 on Serum Glucose Level

A total of 324 shrimp, with an average weight of 10.09 ± 4.08 g, were equally divided into the PBS group, dsEGFP group, and dsRTK2 group, each receiving injections of 10 µL PBS, 10 µL dsEGFP (4 µg/10 µL), and 10 µL dsLvRTK2 (4 µg/10 µL), respectively. After 48 h interference, a glucose solution at a concentration of 1.0 g/kg body weight was injected. Blood samples were collected at 0, 15, 30, 60, 180, and 360 min post-injection. Six shrimp from each group were sampled at each time point, and three biological replicates were collected. Blood was centrifuged (1000 rpm, 4 °C, 5 min), and the upper serum was used to measure glucose concentration using a glucose detection kit (F006-1-1, Jiancheng Bioengineering Institute, Nanjing, China). To present the results visually, the glucose concentration at the initial time was normalized to 100%.

### 2.13. Measurement of Glucose Levels and Enzyme Activity in Hepatopancreas after Interference

Using the long-term interference hepatopancreas collected in Section 2.11 as material, the glucose levels in the hepatopancreas of the interference group and the control group were measured using a glucose detection kit (F006-1-1, Jiancheng Bioengineering Institute, Nanjing, China). In addition, the activity differences of three glucose metabolic enzymes between the two groups were measured, including Hexokinase (HK), Phosphofructokinase (PFK), and Phosphoenolpyruvate carboxykinase (PEPCK). The kits used were purchased from the Nanjing Jiancheng Bioengineering Institute, with product numbers A077-3-1 (HK), A129-1-1 (PFK), and A131-1-1 (PEPCK). The experimental procedures were conducted according to the manufacturer’s protocol.

### 2.14. Measurement of Downstream Gene Expression in Hepatopancreas after Interference

Further analysis was conducted to examine the expression of downstream genes after long-term interference. A total of 15 genes were tested, including the following genes: Protein kinase B (AKT), Glucose-6-phosphate isomerase (G6PI), Glucose transporter (GLUT), Glycogen synthase (GYS), Pyruvate dehydrogenase kinase (PDK1), Phosphoglycerate kinase (PGK), Phosphoinositide-3-kinase (PI3K), Pyruvate kinase (PK), Fructose bisphosptase (FBP), Forkhead box transcription factor (FOXO), Glucose-6-phosphatase (G6PC), Glycogen synthase kinase (GSK), Pyruvate carboxylase (PC), HK, and PEPCK. The 15 genes can be classified into five categories based on their roles in the insulin signaling pathway and downstream glucose metabolism processes. These categories include the insulin downstream phosphatidylinositol 3-kinase/protein kinase B signaling pathway (PI3K, PDK1, Akt, and FOXO), glycolysis (HK, G6PI, PGK, and PK), gluconeogenesis (G6PC, FBP, PEPCK, and PC), glucose transport (GLUT), glycogen synthesis (GYS), and glycogenolysis (GSK). Total RNA from the hepatopancreas was extracted from the interference group and the control group, and cDNA was generated through reverse transcription. The expression of downstream genes was detected using qRT-PCR. The primers used in the experiment are listed in Table 1. The experiment included three biological replicates and three technical replicates, and the results are presented as the mean ± SD.

### 2.15. Statistical Analysis

All experiments were repeated in triplicates and analyzed using SPSS 16.0 (SPSS Inc., Chicago, IL, USA). The results are expressed as mean ± SD. Significant differences (*p* < 0.05) and extremely significant differences (*p* < 0.01) between values were tested using one-way analysis of variance (ANOVA) and assessed using Duncan’s test.

## 3. Results

### 3.1. Molecular Identification of a Novel Insulin-like Receptor (IR) in the L. vannamei

**Figure 1:**
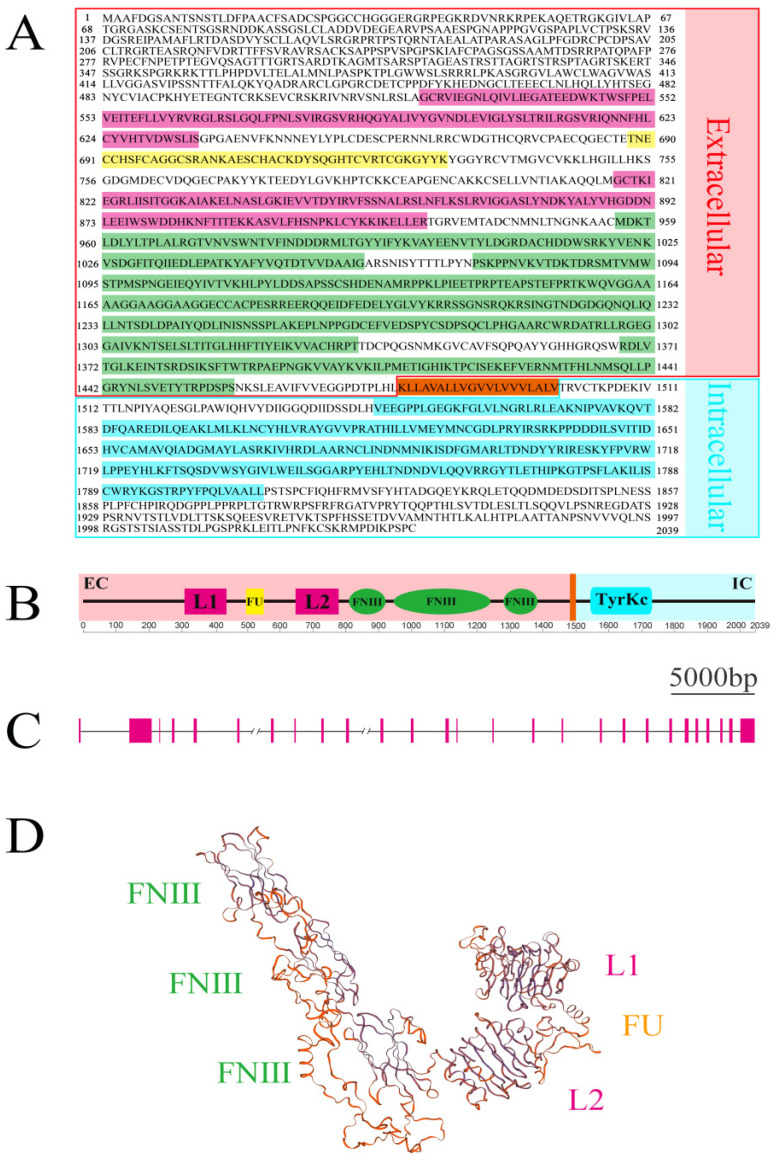
Amino acid sequence and structural prediction of *LvIR*. (**A**) The derived amino acid sequence of *LvIR*, including two ligand-binding domains (highlighted with a purple background), one furin-like domain (yellow background), three FNⅢ domains (green background), one transmembrane domain (orange background), and one tyrosine kinase domain (blue background). The intracellular and extracellular regions are emphasized by blue and pink boxes, respectively. (**B**) Functional domain prediction of *LvIR*. The colors of the functional domains and transmembrane region are consistent with those in (**A**). (**C**) Exon-intron diagram of *LvIR* DNA. Pink boxes represent exons, and black horizontal lines represent introns. Double slashes indicate an unknown length. (**D**) Three-dimensional protein model of the extracellular region of *LvIR*. The locations of the corresponding functional domains are indicated.

Based on the genomic and transcriptomic data of *L. vannamei*, we designed primers targeting the IR. Through multiple rounds of PCR amplification, TA-cloning, and sequencing, we obtained a sequence with a total length of 6439 base pairs (bp). ORFfinder prediction indicated that this sequence contains a 6120 bp open reading frame (ORF), a 126 bp 5′-untranslated region (UTR), and a 193 bp 3′-UTR. The deduced amino acid sequence encodes 2039 amino acid residues with a molecular weight (MW) of 224.52 kDa and a theoretical isoelectric point (pI) of 8.17 (Figure 1A). This sequence shows high similarity to the predicted IR sequences of *Penaeus monodon* and *Penaeus chinensis* in the NCBI database (XP_037791580.1 and XP_047487093.1), with sequence identities of 87.26% and 85.16%, respectively. However, it shares only 34.02% sequence identity with the previously reported IR sequence of *L. vannamei* (XP_027207730.1), indicating that they represent different sequences. These results suggest that we may have discovered a novel IR sequence in *L. vannamei*, which we tentatively name *LvIR*.

The transmembrane domain prediction results indicate that *LvIR* is a single-pass transmembrane protein with its transmembrane region located between amino acids (aa) 1481–1499. According to the SMART domain prediction, the deduced amino acid sequence of *LvIR* contains the following functional domains: two ligand-binding domains (L1: aa 523–635, L2: aa 817–933), one furin-like (FU) domain (aa 688–731), three fibronectin type III (FNIII) domains (aa 956–1059, 1074–1336, and 1368–1458), and an intracellular tyrosine kinase (TyrKc) domain (aa 1548–1807) (Figure 1B). These are typical domains of IR. Additionally, by aligning the *LvIR* sequence with the genome sequence of *L. vannamei*, we obtained its intron and exon information. The results show that its DNA length exceeds 42 kb, containing at least 27 exons and 26 introns (Figure 1C). The three-dimensional model prediction of *LvIR* protein structure shows that the external structure of *LvIR* is an inverted “V” shape, with the L1, Fu, and L2 domains forming one side and the linear arrangement of FNIII domains forming the other side (Figure 1D), which is also a typical feature of IR. Further comparison of the *LvIR* sequence with the *L. vannamei* genome sequence revealed that its sequence in the NCBI database is divided into four segments, including XM_027362264.1, XM_027370182.1, XM_027362239.1, and XM_027362303.1. These sequences all possess partial features of IR, possibly due to incomplete sequencing assembly. Based on the above results, we further confirmed the acquisition of a novel *L. vannamei* IR sequence. Moreover, based on its sequence characteristics, *LvIR* should belong to the tyrosine kinase receptor (RTK)-type IR.

### 3.2. Phylogenetic Analysis

**Figure 2:**
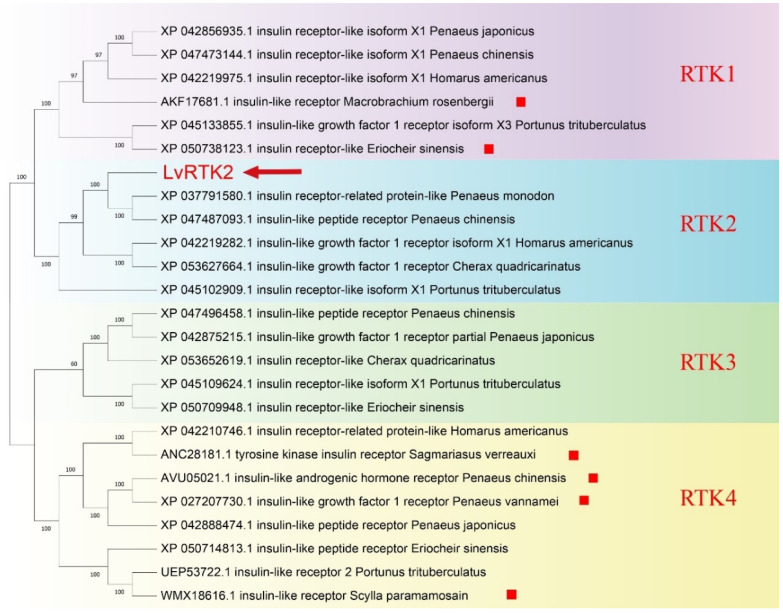
Phylogenetic relationships of insulin-like receptors in decapoda crustacean. The numbers on the forks are the bootstrap proportions for each branch. The red arrow indicates *LvRTK2* found in this study, and the red boxes represent the insulin-like receptors that have been reported.

We collected reported RTK-type IRs of decapod crustaceans and obtained the predicted IR sequences from NCBI. Together with the *LvIR* obtained in this study, we constructed a phylogenetic tree. The results of the phylogenetic tree showed that all IRs were divided into four major categories: RTK1-4, consistent with Veenstra’s previous report in 2020 [20]. The *LvIR* obtained in this study was classified into the second RTK category. Referring to Veenstra’s classification, we officially named our gene as *LvRTK2*. The results show that *LvRTK2* belongs to a different type of IR compared to the previously reported RTK1 type and RTK4 type IR (highlighted in red boxes in Figure 2), further confirming that we have obtained a novel IR sequence. We have uploaded this new sequence to the NCBI database with the accession number PP932464.1.

### 3.3. Multiple Sequence Alignment and Motif Analysis of LvRTK2

**Figure 3:**
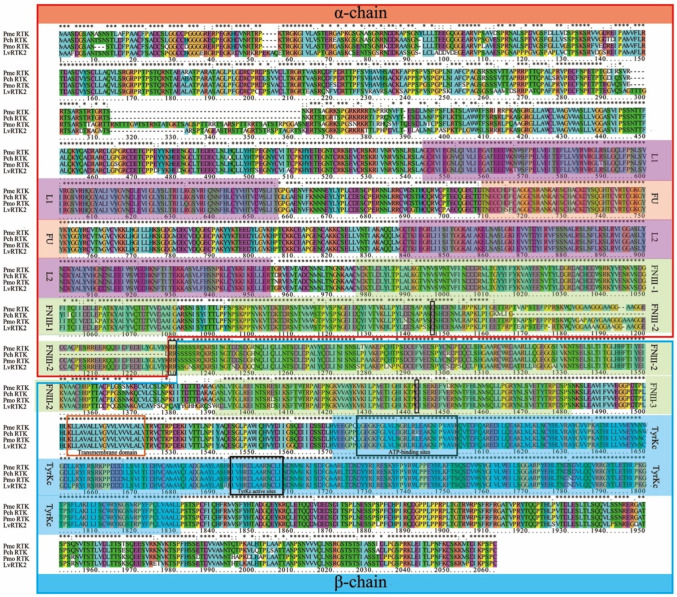
Multiple sequence alignment of *LvRTK2* with other decapoda RTK2 proteins. The IR sequences of *Penaeus merguiensis* (*Pme*), *Penaeus chinensis* (*Pch*), and *Penaeus monodon* (*Pmo*) are sourced from sequences predicted by NCBI. The asterisk (*) indicates conserved amino acids, the colon (:) indicates amino acids with conserved physicochemical properties, and the full stop (.) represents amino acids with weakly similar properties. The α chain, β chain, cleavage sequence, linked cysteine sites, transmembrane domain, ATP-binding site, and tyrosine kinase catalytic activity site are highlighted in different colors and boxes. Annotations are marked on the sides or below the figure.

The predicted IR sequences from other decapoda species (*Penaeus merguiensis*, *Penaeus chinensis*, and *Penaeus monodon*) were obtained from NCBI and aligned with the *LvRTK2* sequence for multiple sequence comparison. The results showed that RTK2 is highly conserved at the sequence level among decapod species, especially in critical functional domains like the L1/L2, FU, FNIII, and TyrKc domains (Figure 3).

In addition, the functional motifs of *LvRTK2* were predicted and annotated in this study, and these sites were found to be highly conserved among decapod species (Figure 3). Specifically, *LvRTK2* is composed of two subunits (α chain: aa 1–1206 and β chain: aa 1207–2039) separated by a conserved “RR” sequence (aa 1206–1207) as the cleavage site. Moreover, the α and β chains are stabilized by disulfide bonds, and the predicted disulfide bond linking the two subunits is formed by Cys1125 in FNIII-2 and Cys1419 in FNIII-3. The transmembrane domain is located in the β chain after FNIII-3, spanning 19 amino acids (aa 1481–1499). The TyrKc domain is located in the intracellular portion of the β chain, containing a tyrosine kinase catalytic activity site defined by the sequence IVHRDLAARNCLI (aa 1672–1684) and an ATP binding site (LGEGKFGLVLNGRLRLEAKNIPVAVK, aa 1554–1579). Additionally, it is predicted that there are 14 glycosylation sites and 8 phosphorylation sites in the extracellular and intracellular regions of *LvRTK2*, respectively. The multiple sequence alignment results show that these sites are also highly conserved among different decapod species (Figure S1).

### 3.4. Spatial and Temporal Expression Profile of LvRTK2

**Figure 4:**
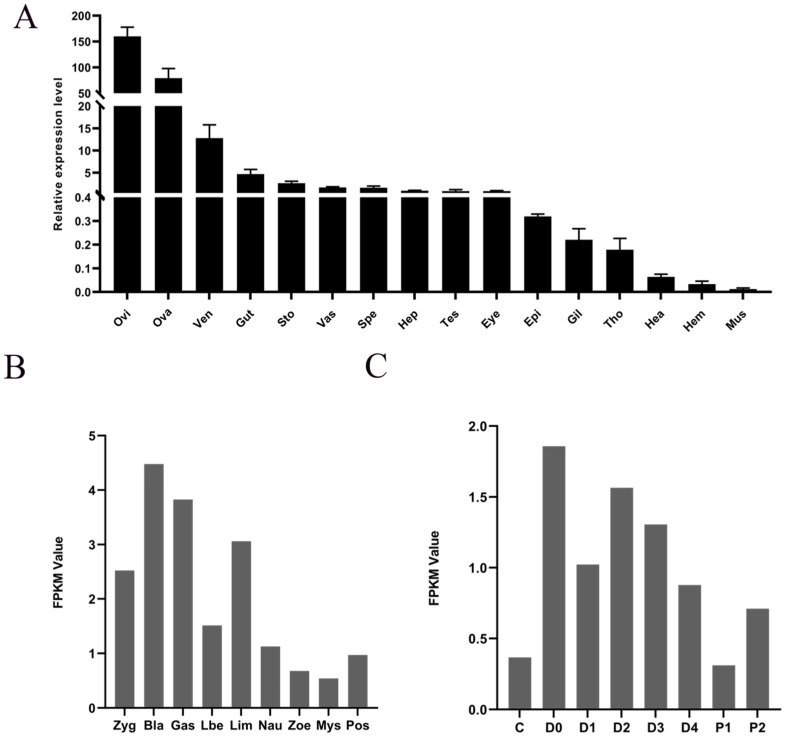
Spatial and temporal expression profiles of *LvRTK2*. (**A**) Relative expression levels of *LvRTK2* in sixteen adult tissues, including oviduct (Ovi), ovary (Ova), ventral nerve cord (Ven), gut (Gut), stomach (Sto), vas deferens (Vas), sperm atophore (Spe), hepatopancreas (Hep), testis (Tes), eyestalk (Eye), epidermis (Epi), gill (Gil), thoracic ganglia (Tho), heart (Hea), hemocyte (Hem), and muscle (Mus). The number of biological replicates per tissue is six, with three biological replicates (n = 18). The error bars for each column represent standard deviations. (**B**) FPKM value of *LvRTK2* at nine early developmental stages, including the zygote (Zyg), blastula (Bla), gastrula (Gas), limb bud embryo (Lim), nauplius (Nau), zoea (Zoe), mysis (Mys), and post-larvae (Pos) stages. (**C**) FPKM value of *LvRTK2* at eight molting stages, including the inter-molt (C stage), pre-molt (D0, D1, D2, D3, and D4 stages), and post-molt (P1 and P2 stages) stages.

The expression profile of *LvRTK2* across 16 adult tissues was detected using qRT-PCR. The results showed significant differences in *LvRTK2* gene expression levels among different tissues, with the highest expression levels found in the oviduct and ovary, followed by the ventral nerve cord, gut, stomach, vas deferens, sperm atophore, hepatopancreas, testis, and eyestalk, while the expression levels in the remaining tissues were relatively low (Figure 4A).

The expression profile of *LvRTK2* at nine embryonic/larval stages and eight molting stages was derived from the newly assembled transcriptomic data. Reference genes are based on the latest version of the *L. vannamei* genome from NCBI, and the newly obtained *LvRTK2* sequences were used to replace four fragments (XM_027362264.1, XM_027370182.1, XM_027362239.1, and XM_027362303.1) for the most accurate expression results. The results showed that the expression of *LvRTK2* was mainly concentrated in the early embryonic stages, from the zygote to the larva in the membrane (Lim) stage. Once hatched into larvae, *LvRTK2* expression significantly decreased (Figure 4B). Additionally, during different molting stages, *LvRTK2* expression was mainly concentrated in the pre-molt (D0, D1, D2, D3, and D4) stages, significantly higher than in the inter-molt and post-molt stages (Figure 4C).

### 3.5. In Vivo and In Vitro Glucose Treatment Experiments on LvRTK2 Gene Expression

**Figure 5:**
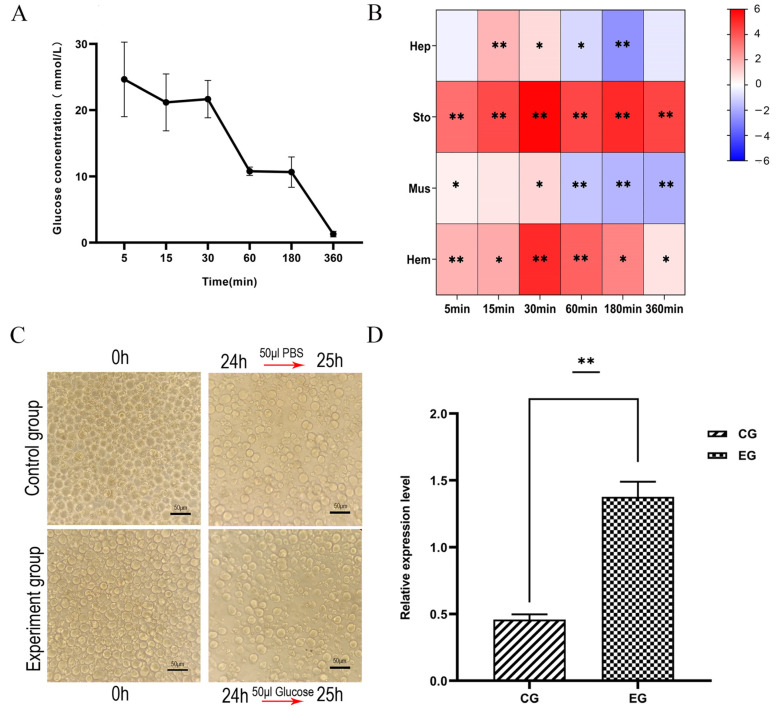
Effects of glucose treatment on glucose levels and *LvRTK2* expression. (**A**) Changes in serum glucose concentration after in vivo glucose injection. (**B**) Log_2_ fold changes of *LvRTK2* expression between the experimental and control groups after glucose injection. Red indicates upregulation and blue indicates downregulation. (**C**) Images of primary hepatopancreatic cells treated. (**D**) Changes in *LvRTK2* expression under in vitro glucose treatment between the control group (CG) and experimental group (EG). Error bars represent standard deviation. Statistical significance is indicated as * *p* < 0.05 and ** *p* < 0.01.

By injecting a high concentration of glucose solution into shrimp, the glucose concentration in the shrimp serum increased to 24.64 mmol/L within 5 min. Subsequently, the concentration continuously decreased, dropping to 1.29 mmol/L at 360 min (Figure 5A). During the gradual clearance of glucose in vivo, the expression of *LvRTK2* significantly increased in all tissues examined. Specifically, in the hepatopancreas and muscle, the up-regulation of *LvRTK2* mainly occurred in the early stages (5 min, 15 min, and 30 min), whereas, in stomach and hemocytes, the *LvRTK2* expression was significantly induced throughout the entire 6 h (Figure 5B).

Additionally, we cultured primary hepatopancreatic cells of *L. vannamei* to further validate the effect of glucose addition on *LvRTK2* gene expression through in vitro experiments. The results indicated that the expression of *LvRTK2* significantly increased in hepatopancreas cells treated with glucose solution compared to the control group, with the expression level in the experimental group being three times higher than that in the control group (Figure 5D).

### 3.6. In Vivo and In Vitro Exogenous Insulin Treatment Experiments on LvRTK2 Gene Expression

To further validate the role of *LvRTK2* in the glucose metabolism process in shrimp, we chose bovine insulin injection as an exogenous insulin stimulus to observe the changes in shrimp blood glucose levels and the expression of the *LvRTK2* gene. Studies have shown that bovine insulin, as an exogenous source of insulin, can cause fluctuations in blood glucose levels in crustaceans and insects [46,47].

**Figure 6:**
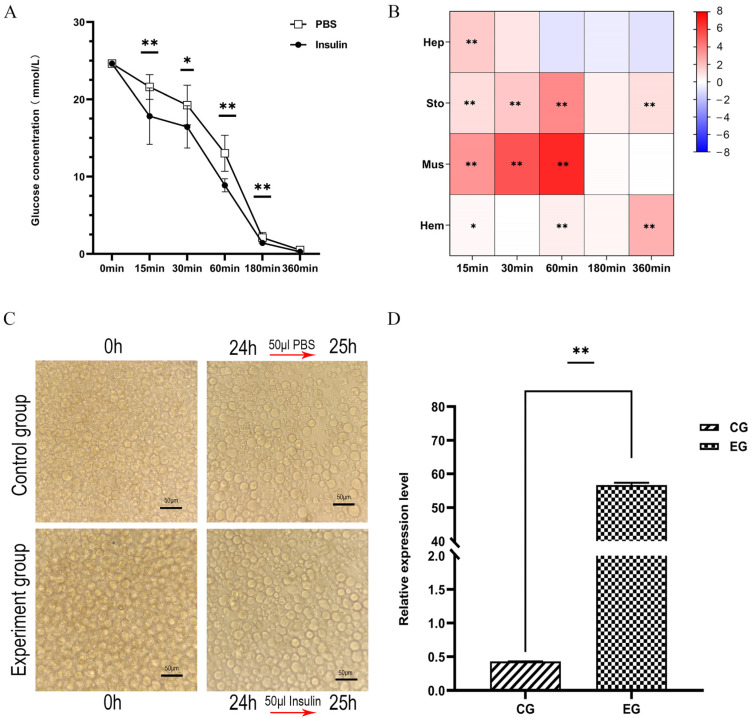
Effects of exogenous insulin treatment on glucose levels and *LvRTK2* expression. (**A**) Changes in serum glucose concentration after in vivo exogenous insulin injection. (**B**) Log_2_ fold changes of *LvRTK2* expression between the experimental and control groups after exogenous insulin injection. Red indicates up-regulation and blue indicates down-regulation. (**C**) Images of primary hepatopancreatic cells treated. (**D**) Changes in *LvRTK2* expression under in vitro exogenous insulin treatment between the control group (CG) and experimental group (EG). Error bars represent standard deviation. Statistical significance is indicated as * *p* < 0.05 and ** *p* < 0.01.

The results indicated that the injection of exogenous insulin accelerated the clearance rate of glucose in the serum of the experimental group. After the injection of the same glucose solution, the serum glucose level in the experimental group that received exogenous insulin decreased significantly faster than that of the control group. It was not until 6 h later that there was no significant difference observed between the two groups (Figure 6A). Additionally, we measured the changes in *LvRTK2* gene expression during this process, and the results showed a significant up-regulation of *LvRTK2* expression in all four tissues, especially in the stomach, muscle, and hemocyte (Figure 6B).

Moreover, in vitro experiments demonstrated that adding exogenous insulin as a supplement to primary hepatopancreatic cells led to a significant up-regulation of *LvRTK2* expression. Compared to the control group, the expression level of *LvRTK2* in the experimental group was 131.81 times higher than that in the control group (Figure 6D).

### 3.7. In Vivo and In Vitro Starvation Treatment Experiments on LvRTK2 Gene Expression

**Figure 7:**
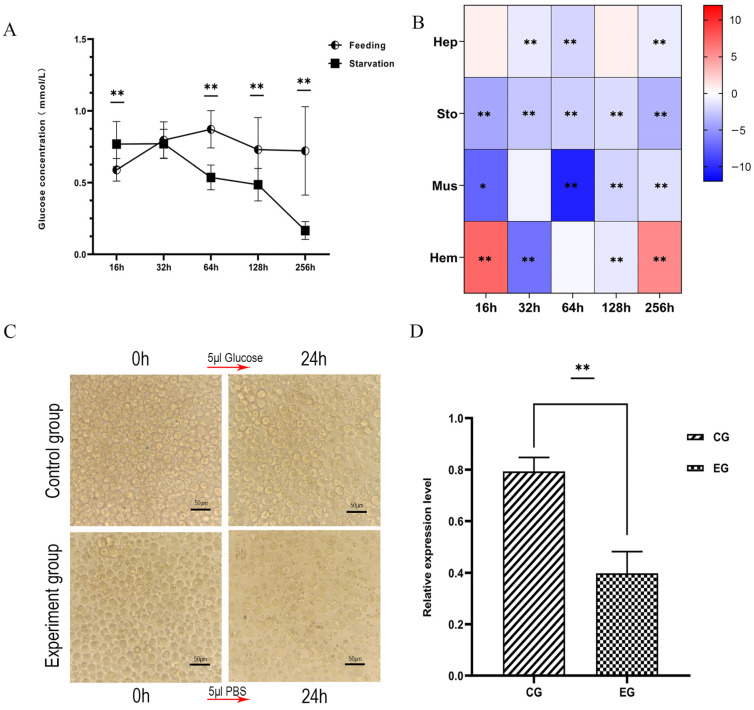
Effects of starvation treatment on glucose levels and *LvRTK2* expression. (**A**) Changes in serum glucose concentration after in vivo starvation treatment. (**B**) Log_2_ fold changes of *LvRTK2* expression between the experimental and control groups after starvation treatment. Red indicates up-regulation and blue indicates down-regulation. (**C**) Images of primary hepatopancreatic cells treated. There is a significant decrease in cell density in the experimental group compared to the control group after 24 h of treatment. (**D**) Changes in *LvRTK2* expression under in vitro starvation treatment between the control group (CG) and experimental group (EG). Error bars represent standard deviation. Statistical significance is indicated as * *p* < 0.05 and ** *p* < 0.01.

We further selected starvation treatment to verify the changes in glucose levels in shrimp and the corresponding gene changes of *LvRTK2*. In the in vivo experiment, the starvation treatment lasted a total of 256 h, during which the control group was fed normally. The results showed that, under starvation treatment, the serum glucose levels in the experimental group initially increased within 16 h, and then continued to decline after 32 h. The serum glucose levels of the experimental group began to be significantly lower than those of the control group after 32 h. By the end of 256 h, the serum glucose concentration in the experimental group dropped to 0.17 mmol/L, a decrease of 77.92% compared to the value at 16 h (Figure 7A). Correspondingly, the expression of *LvRTK2* in the hepatopancreas and hemocyte initially increased at 16 h and then decreased. In the stomach and muscle tissues, *LvRTK2* exhibited significant down-regulation at all sampling time points (Figure 7B).

In the in vitro experiments, the sugar source in the culture medium of primary hepatopancreas cells was replaced to simulate starvation treatment. The results showed that, firstly, after 24 h of treatment, the cell density in the experimental group significantly decreased to a point that was markedly lower than that of the control group, likely due to the effects of starvation treatment (Figure 7C). Additionally, starvation treatment significantly down-regulated the expression of *LvRTK2*, with the expression level of *LvRTK2* in the experimental group reduced by 49.37% compared to the control group (Figure 7D).

### 3.8. Long-Term RNA Interference Experiment of LvRTK2

**Figure 8:**
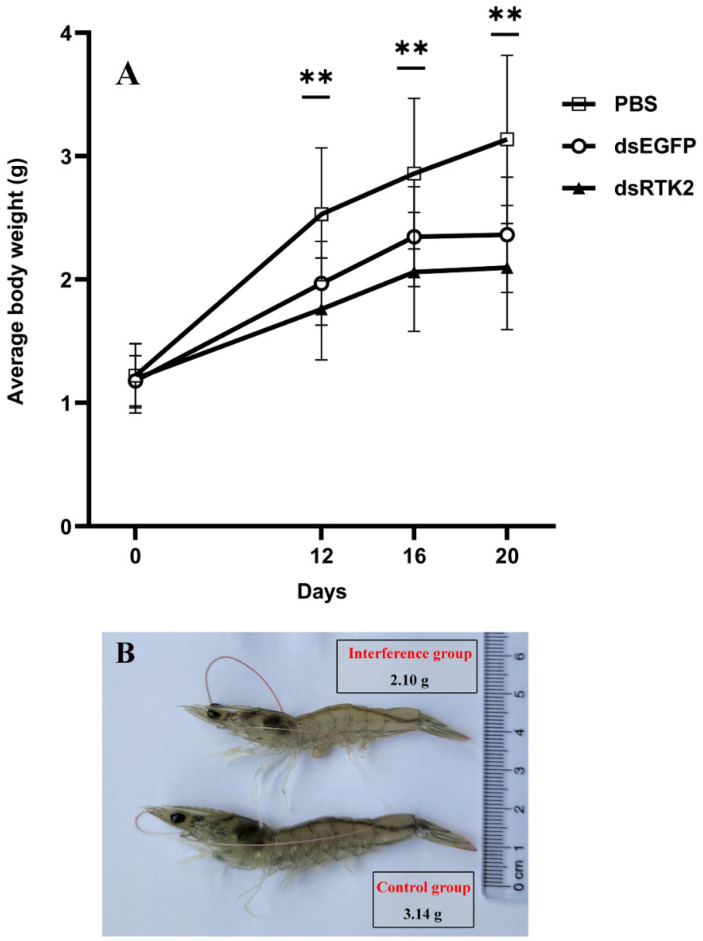
Measurement of shrimp body weight under long-term *LvRTK2* interference. (**A**) Results of average body weight measurement. The asterisk symbol (**) indicates that there are significant differences between the dsRTK2 group and both the dsEGFP group and the PBS group (*p* < 0.01). (**B**) Representative photo showing differences in shrimp size between the experimental group and the control group after 20 days interference. The selected shrimp represent the average body weight. Error bars indicate standard deviation, with statistical significance as ** *p* < 0.01.

A long-term interference experiment targeting *LvRTK2* was conducted to investigate its effect on the growth of *L. vannamei*. The experiment lasted for 20 days, during which dsLvRTK2 reagent was injected every 4 days. From day 12 onwards, the average weight changes of the shrimp were recorded. The results demonstrated that, compared to the dsEGFP group and the control (PBS) group, the interference with *LvRTK2* significantly reduced the growth rate of the shrimp (Figure 8A). Starting from day 12, the average weight of the shrimp in the experimental group was significantly lower than that of the control group and dsEGFP group (*p* < 0.01). After 20 days of interference, the average weight of the control group (3.14 ± 1.44 g) was 1.50 times that of the interference group (2.10 ± 0.90 g). The significant difference in individual size between the two groups can be clearly observed (Figure 8B).

### 3.9. Short-Term RNA Interference Experiment of LvRTK2

**Figure 9:**
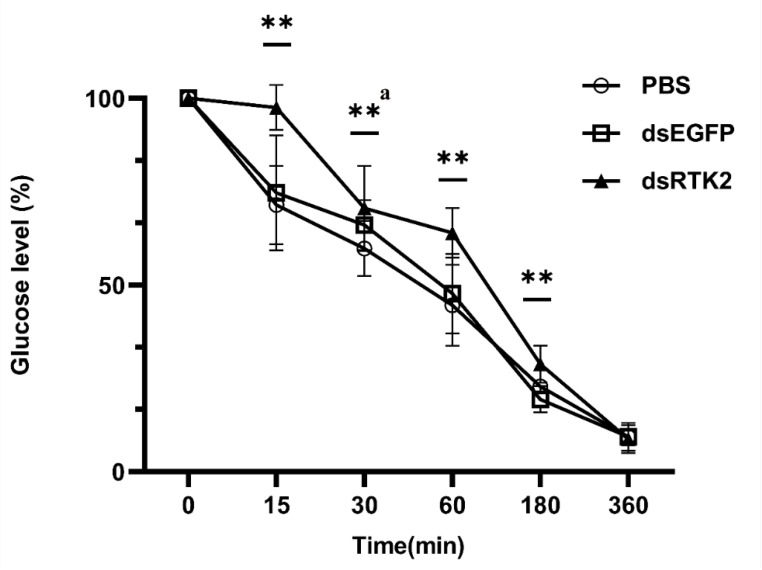
Measurement of serum glucose levels under short-term *LvRTK2* interference. To present the results visually, the glucose concentration at the initial time is normalized to 100%, and the data for the remaining time points are correspondingly homogenized. Error bars represent the standard deviation. The statistical significance was ** *p* < 0.01 (where ‘a’ indicates that the dsRTK2 group data only show a significant difference compared to the control group).

To understand the reasons behind the weight loss in shrimp caused by the long-term interference of *LvRTK2*, we subsequently conducted a short-term interference experiment targeting *LvRTK2*. After 48 h of RNA interference, glucose solution (1.0 g/kg body weight) was injected into the shrimp to observe the glucose metabolism capability of *L. vannamei* following the knockdown of *LvRTK2*. The results showed that the serum glucose clearance rate in the dsLvRTK2 group was significantly lower than that in the control group and the dsEGFP group. Notably, at 15 min post-injection, the serum glucose level in the experimental group was almost the same as the initial state, at 97.51% of the initial glucose level. In contrast, the serum glucose levels in the control group and dsEGFP group significantly decreased, reaching only 71.40% and 74.67% of their initial levels, respectively (Figure 9). It was not until 6 h post-injection that there were no significant differences in serum glucose levels among the experimental group, control group, and dsEGFP group.

### 3.10. Effect of Lv-RTK2 Silencing on Glucose Levels and Enzyme Activity in the Hepatopancreas

**Figure 10:**
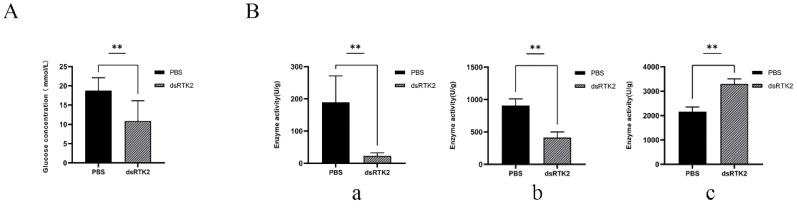
Measurement of glucose concentration and enzyme activity in the hepatopancreas after long-term *LvRTK2* interference. (**A**) Changes in glucose concentration in hepatopancreas between the control group (PBS) and the interference group (dsRTK2). (**B**) Differences in the activity of glucose metabolism-related enzymes. (**a**): HK, (**b**): PFK, and (**c**): PEPCK. Error bars represent standard deviation. The statistical significance was ** *p* < 0.01.

To further understand the reasons behind the weight loss in shrimp caused by *LvRTK2* interference, we measured the glucose content and the enzyme activities related to glucose metabolism in the hepatopancreas of the interference group and the control group after long-term interference. The results showed that the glucose level in the hepatopancreas of the long-term interference group was only 58.08% of that in the control group (Figure 10A). Meanwhile, the activities of the key limiting enzymes related to glycolysis, HK and PFK, were significantly down-regulated in the interference group, while the activity of the gluconeogenesis-related enzyme PEPCK was significantly up-regulated. Specifically, the enzyme activities of HK and PFK were down-regulated by 87.74% and 54.64%, respectively, while the enzyme activity of PEPCK was up-regulated by 49.57% (Figure 10B).

### 3.11. Effect of LvRTK2 Silencing on Downstream Pathways and the Expression of Genes Related to Glucose Metabolism

**Figure 11:**
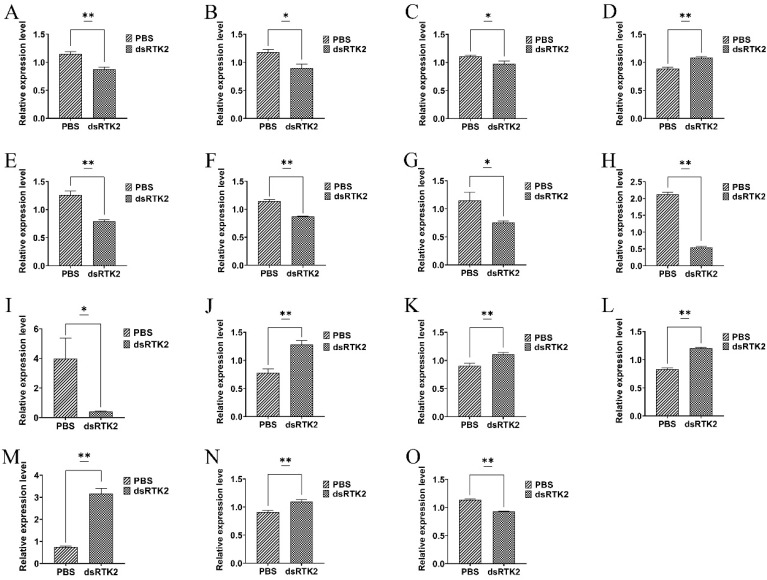
Measurement of downstream gene expression in the hepatopancreas after long-term *LvRTK2* interference. The changes in relative expression levels of genes including (**A**): PI3K, (**B**): PDK1, (**C**): AKT, (**D**): FOXO, (**E**): GLUT, (**F**): HK, (**G**): G6PI, (**H**): PGK, (**I**): PK, (**J**): G6PC, (**K**): FBP, (**L**): PEPCK, (**M**): PC, (**N**): GSK, and (**O**): GYS were measured. Error bars refer to the standard deviation. Statistical significance is marked as * *p* < 0.05 and ** *p* < 0.01.

**Figure 12:**
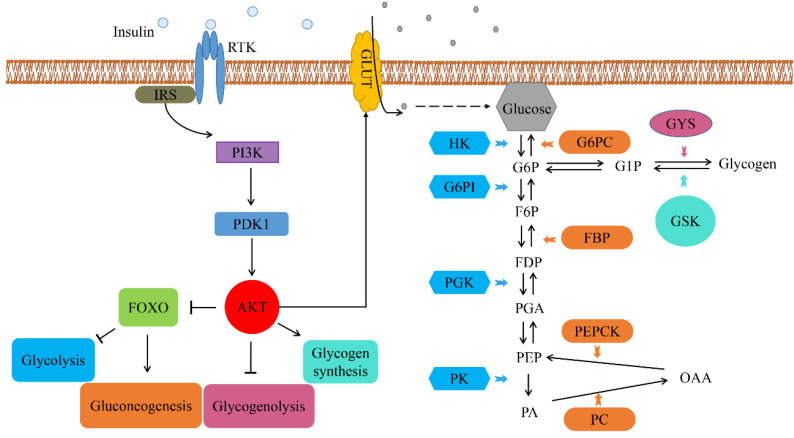
Schematic pathway diagram of receptor tyrosine kinase (RTK) regulating the growth and glucose metabolism of *L. vannamei*. The pathway is drawn based on the Kyoto Encyclopedia of Genes and Genomes (KEGG) database (map04910) and our research findings. Lines ending with an arrow indicate promoting effects, while lines ending with a flat line indicate inhibitory effects. G6P: Glucose-6-phosphate, G1P: Glucose-1-phosphate, F6P: Fructose 6-Phosphate, FDP: Fructose Diphosphate, PGA: Phosphoglycerate, PEP: Phosphoenolpyruvate, PA: Pyruvic acid, OAA: Oxaloacetic acid.

We focused on studying the effects of *LvRTK2* silencing on gene expression related to the insulin downstream signaling pathway and a series of genes associated with glucose metabolic events, including genes associated with glucose transport, glycolysis, gluconeogenesis, glycogen synthesis, and glycogenolysis. A total of 15 gene expressions were measured. The results showed that, after long-term interference with *LvRTK2*, the expression of key genes in the insulin downstream PI3K/Akt signaling pathway was significantly down-regulated in the experimental group, with the expressions of PI3K, PDK1, and AKT down-regulated by 24.35%, 23.73%, and 11.82%, respectively (Figure 11A–C). In contrast, the expression of FOXO was up-regulated by 21.35% (Figure 11D). The expression of the glucose transporter GLUT was down-regulated by 37.30% (Figure 11E). Moreover, the expressions of key genes in the glycolysis pathway, including HK, G6PI, PGK, and PK, were down-regulated by 24.35%, 34.78%, 74.65%, and 89.42%, respectively (Figure 11F–I). On the other hand, after interference, the expressions of key genes in the gluconeogenesis pathway, including G6PC, FBP, PEPCK, and PC, were up-regulated by 64.10%, 21.98%, 45.78%, and 328.38% respectively (Figure 11J–M). Additionally, the gene responsible for glycogenolysis, GSK, was activated (Figure 11N), with its expression up-regulated by 20.88%. In contrast, the expression of the gene involved in glycogen synthesis, GYS, was down-regulated by 18.42% (Figure 11O). To clarify the impact of *LvRTK2* on the biological processes of these genes, we speculated and plotted the downstream gene signaling pathway of *LvRTK2* (Figure 12).

## 4. Discussion

### 4.1. Discovery and Sequence Analysis of LvRTK2

The insulin signaling pathway is a conserved regulatory pathway that is widely present in Metazoans [48]. It is activated by the interaction of insulin/insulin-like peptides (ILPs) with insulin receptors/insulin-like receptors (IRs) and is responsible for regulating various biological functions [4,5]. In mammals and insects, the members of the insulin signaling pathway and their functions have been well elucidated [4,6,7]. However, research on the insulin signaling pathway in crustaceans remains limited. In fact, our understanding of the members of the crustacean insulin signaling pathway has gradually deepened with the recent development of sequencing technology [36,49,50]. For instance, it was not until 2015 that the second type of ILP, known as DILP7/relaxin type ILP, was discovered in crustaceans [51]. Prior to this, it was believed that crustaceans only had one type of ILP, known as IAG [52]. Furthermore, it was not until 2020 that the existence of four types of RTKs in decapod crustaceans was established [20], rather than just RTK4. This has also raised questions about the characteristics and functions of the other three classes of RTKs (RTK1-3). However, the current research on these other three classes of RTKs is still very limited, and even the reported sequences of RTKs are fragmented. Taking the *LvRTK2* sequence obtained in this study as an example, its original sequence is divided into four fragments on NCBI, including XM_027362264.1, XM_027370182.1, XM_027362239.1, and XM_027362303.1.

Therefore, the first major contribution of this study is the first acquisition of the complete sequence of the RTK2 gene in *L. vannamei*, confirming its integrity rather than four independent segments. The full length of *LvRTK2* is 6439 bp, which poses a challenge in obtaining its complete sequence. The newly obtained sequence of *LvRTK2* has also been submitted to NCBI (GenBank accession number: PP932464.1), and we recommend using this new *LvRTK2* sequence instead of the four fragmented sequences in future studies. Additionally, it is worth noting that this study not only discovered and cloned the *LvRTK2* gene sequence for the first time but also provided a comprehensive description of the sequence features, sequence structure, and gene expression characteristics of the *RTK2* gene in *L. vannamei*.

#### 4.1.1. Sequence Features Analysis

The ORF sequence length of *LvRTK2* is 6120 bp, which appears to be the longest ORF among the reported crustacean RTKs. Its length exceeds that of the reported RTK4-type IRs, such as *FcRTK4* 5436 bp (*F. chinensis*), *SvRTK4* 6018 bp (*S. verreauxi*), *LvRTK4* 5490 bp (*L. vannamei*), and *SpRTK4* 5673 bp (*S. paramamosain*) [21,22,23,24], as well as RTK1-type IRs, such as *MrRTK1* 4527 bp (*M. rosenbergii*) and *EsRTK1* 4326 bp (*E. sinensis*) [27,28]. Furthermore, *LvRTK2*’s length exceeds that of IRs in other species, such as *HsIR* 4155 bp (*Homo sapiens*), *BmIR* 4419 bp (*Bombyx mori*), *TcIR* 2760 bp (*Tribolium castaneum*), *DrIR* 4047 bp (*Danio rerio*), *LmIR* 4077 bp (*Locusta migratoria*), *DpIR* 4509 bp (*Daphnia pulex*), and *CeIR* 5541 bp (*Caenorhabditis elegans*) [15,53,54,55].

#### 4.1.2. Sequence Structure Analysis

It is noteworthy that, despite differences in sequence length, almost all identified IRs exhibit a highly conserved structure. This includes the reported RTK1 and RTK4 in crustaceans, as well as the RTK2 studied in this research. They all consist of two ligand-binding (L1 and L2) domains, a cysteine-rich domain, three fibronectin III (FN3) domains, a transmembrane domain, and an intracellular tyrosine kinase (TyrKc) domain [21,22,23,24,26]. Furthermore, these structural units are conserved across evolution, from invertebrates to vertebrates [27,56], demonstrating the highly conserved sequence structure of IRs in metazoans.

Additionally, several conserved motifs have also been identified in the RTK2 sequence of *L. vannamei*, such as the α and β chain cleavage site, transmembrane domain site, ATP binding site, tyrosine kinase site, glycosylation sites, and phosphorylation sites. These motif sequences are mostly predicted and reported for the first time in crustaceans, such as the conserved “RR” sequence (aa 1206–1207) as the cleavage site, the disulfide bond formation site formed by Cys residues (aa 1125–1419), and glycosylation/phosphorylation sites. Additionally, the three-dimensional prediction model of the extracellular region of *LvRTK2* protein exhibited a highly conserved inverted “V” shape, similar to that of the IR in humans [57]. All these structures indicate that *LvRTK2* possesses a highly conserved sequence structure.

#### 4.1.3. Gene Expression Pattern Analysis

The tissue expression results of *LvRTK2* show that it is primarily expressed in the female gonad and oviducts. Notably, this expression pattern is completely opposite to that of crustacean RTK4, which is predominantly expressed in the male reproductive organs, such as the sperm atophore and vas deferens, and has been identified as the receptor for IAG [21,23]. This suggests that *LvRTK2* may be involved in the development of female reproductive organs in crustaceans, playing a role in sex determination that is distinct from the function of RTK4 and IAG. Incidentally, we plan to explore the role of RTK2 in sex differentiation in our next study.

In addition to reproductive organs, it has been found that *LvRTK2* is mainly expressed in the digestive organs (intestine, stomach, and hepatopancreas) of *L. vannamei*, indicating its potential involvement in nutrient metabolism. Moreover, transcriptomic data from early developmental stages confirm that *LvRTK2* exhibits higher expression during the zygote stage, indicating its potential as a maternally expressed gene. Additionally, transcriptomic data related to molting reveal that *LvRTK2* is primarily expressed in the pre-molt stage (D0–D4 stage), which may be associated with the accumulation of energy storage and the synthesis of carbohydrates during this molting phase [58].

### 4.2. The Regulatory Role of LvRTK2 in Glucose Metabolism of L. vannamei

A continuous supply of blood glucose ensures the normal function and survival of organisms [59,60]. Under normal circumstances, the concentration of glucose in the bloodstream can be maintained within a certain range, and this plays a crucial role in maintaining the normal physiological activities of animals [1,61].

Although previous studies have indicated the presence of an endogenous insulin signaling pathway involved in carbohydrate metabolism in crustaceans [51,62,63], current evidence regarding the carbohydrate metabolism pathways in crustaceans is relatively weak [34], and there are still many unresolved questions. For example, although the existence of RTKs has been identified in crustaceans, it is still unclear whether they have a similar function in regulating glucose metabolism as vertebrate IRs.

In this study, we provided evidence from both in vivo and in vitro experiments, including glucose treatment, exogenous insulin treatment, and starvation treatment, to establish the relationship between *LvRTK2* and glucose regulation in shrimp. The conclusions include the following:

#### 4.2.1. Glucose Treatment

By injecting shrimp with a high-concentration glucose solution, it was observed that the serum glucose concentration could rise to 24.64 mmol/L within 5 min post-injection. The high glucose concentration could return to baseline levels (~1 mmol/L) within 6 h, further confirming the presence of an endogenous glucose regulation/clearance mechanism in shrimp [46,63]. During this process, the expression of *LvRTK2* significantly increased in all examined tissues. Specifically, in the hepatopancreas and muscle, the up-regulation of *LvRTK2* mainly occurred in the early stage (5 min, 15 min, and 30 min), whereas, in muscle and hemocytes, *LvRTK2* expression was induced within 6 h. At the cellular level, we further confirmed that the addition of glucose solution to the culture medium significantly increased *LvRTK2* expression. These results indicate that shrimp can regulate glucose levels in different tissues in response to exogenous glucose treatment, and *LvRTK2* plays an important role in this process.

#### 4.2.2. Exogenous Insulin Treatment

The results showed that the injection of exogenous bovine insulin accelerated the rate of glucose clearance in the serum of the experimental group. Starting from 15 min after injection, the serum glucose levels in the experimental group were significantly lower than those in the control group, indicating that shrimp could respond to exogenous insulin and regulate glucose levels in the body. This finding is consistent with the results observed in *Drosophila melanogaster* [64], *Callinectes sapidus* [65], and *Neohelice granulata* [66], where exogenous insulin was injected. In this process, *LvRTK2* was significantly up-regulated in all four types of tissues. Experiments with in vitro primary cells also demonstrated that the addition of exogenous insulin could increase the expression of *LvRTK2*. These results suggest that *LvRTK2* is involved in the process of shrimp’s response to exogenous insulin injection/addition and in the regulation of blood glucose levels.

#### 4.2.3. Starvation Treatment

Under starvation treatment, the serum glucose levels of the experimental group increased compared to the control group during the initial 16 h, and then gradually decreased after 32 h, consistent with previous research findings [63]. The initial increase in blood glucose level may be attributed to the shrimp’s stress response to starvation [63], in which glycogen, pyruvate, and amino acids are converted into glucose during the early stages of starvation [67]. However, prolonged starvation eventually depletes these nutrient reserves, leading to an inevitable decrease in serum glucose concentration [68]. Consistent with the changes in serum glucose level, the expression of *LvRTK2* also showed a similar trend, especially in the hepatopancreas and hemocytes, with an initial increase at 16 h followed by a continuous decrease after 32 h. In stomach and muscle tissues, *LvRTK2* was down-regulated at all detected time points. Additionally, in primary hepatopancreatic cells subjected to 24 h of starvation simulation, *LvRTK2* expression was also significantly down-regulated in the experimental group. These results confirm that shrimp have specific glucose regulation strategies in response to starvation [69], and it is hypothesized that *LvRTK2* may play a role in coping with glucose deficiency caused by starvation by regulating the activity of the insulin signaling pathway.

In summary, the second major contribution of this study is clearly demonstrating the existence of a glucose regulation pathway in *L. vannamei*. Whether it is high glucose treatment, exogenous insulin treatment, or starvation treatment, shrimp exhibit corresponding strategies for blood glucose regulation to maintain stable glucose levels, and *LvRTK2* is evidently involved in this series of regulatory processes.

### 4.3. LvRTK2 Regulates the Growth of L. vannamei

Many decapod crustaceans, such as shrimp, lobsters, and crabs, are important economic species, but research on their growth regulation mechanisms remains limited [34]. Increasing evidence indicates a close relationship between the insulin signaling pathway and the growth of crustaceans [52,70]. However, for a long time, research on the insulin signaling pathway in decapod crustaceans has primarily focused on sexual differentiation, represented by research on IAG and RTK4 [21,71]. Recently, more types of insulin-like receptors, such as RTK1-3, have been discovered in decapod crustaceans [20,72], but the understanding of the functions of these RTKs is still limited, especially their role in shrimp growth regulation. In this study, the identification of *LvRTK2* confirmed the presence of a second type of RTK (RTK2) in *L. vannamei* and provided its sequence characteristics (Section 3.1, Section 3.2, Section 3.3 and Section 3.4), as well as an analysis of its role in regulating glucose levels (Section 3.5, Section 3.6 and Section 3.7). Additionally, another major contribution of this study was to provide clear evidence indicating that *LvRTK2* may have an impotent role in regulating the growth of decapod crustaceans, as interfering with *LvRTK2* significantly affected the growth of *L. vannamei* (long-term interference) and the process of glucose regulation (short-term interference). Key conclusions include the following:

First, we conducted a long-term interference experiment on *LvRTK2* using RNA interference (RNAi) technology for a period of 20 days. The results indicated that interfering with *LvRTK2* significantly reduced the growth rate of shrimp. Starting from day 12, the average body weight of the experimental group was significantly lower than that of the control group and the dsEGFP group. After 20 days of interference, the average body weight of the control group was 1.5 times that of the interference group. Incidentally, the growth differences between the dsEGFP group and the control group may be attributed to the RNAi mechanism [73], as the siRNA derived from the degradation of the dsEGFP fragment inevitably interacts with homologous sequences within the shrimp, potentially affecting their growth. However, it can be confirmed that there are significant differences between the dsLvRTK2 group and both the dsEGFP group and the PBS group. For instance, on days 12, 16, and 20 of measurement, the body weights of the dsEGFP group were 1.12, 1.14, and 1.12 times that of the dsLvRTK2 group, respectively, and all data showed statistically significant differences (*p* < 0.01). These results suggest a close correlation between *LvRTK2* and the growth of *L. vannamei*.

To further understand the reasons for the slow growth of shrimp caused by long-term interference of *LvRTK2*, we conducted a short-term RNAi experiment. Forty-eight hours after interfering with the *LvRTK2*, we injected the shrimp with a glucose solution with a concentration of 1 g/kg. The clearance rate of exogenous glucose in the serum was measured within 6 h for the experimental group, the control group, and the dsEGFP group. The results showed that the clearance rate of exogenous glucose in the serum of the interference group was significantly lower than that of the other two groups. This indicates that the interference with *LvRTK2* gene expression significantly affects glucose utilization in shrimp. It is speculated that the ILP in the interference group cannot initiate the insulin signaling pathway through RTK, thereby preventing glucose from entering the cells and leading to insufficient intracellular glucose levels. Consequently, the shrimp lack adequate glucose for metabolism and growth. This may be the primary reason for the reduced growth rate caused by the interference of *LvRTK2*.

To verify the speculation, we measured the glucose content in the hepatopancreas of the shrimp after 20 days of long-term interference. As expected, we found that the glucose content in the hepatopancreas of the interference group was significantly lower than that of the control group. This indicates that interference with *LvRTK2* affected glucose entry into the cells and tissues, confirming the hypothesis that *LvRTK2* RNAi leads to reduced glucose content in the cells.

Next, to further explain the underlying mechanisms causing slow growth in shrimp after *LvRTK2* interference, we measured the enzyme activities of key enzymes involved in glucose metabolism in the hepatopancreas after long-term interference. These enzymes include hexokinase (HK), phosphofructokinase (PFK), and phosphoenolpyruvate carboxykinase (PEPCK), which are critical rate-limiting enzymes in glycolysis and gluconeogenesis [74]. The results showed that the enzyme activities of the glycolytic key enzymes, HK and PFK, were significantly down-regulated after *LvRTK2* interference, while the enzyme activity of the gluconeogenic key enzyme, PEPCK, was significantly up-regulated. This reflects the response of the shrimp to *LvRTK2* interference. It is speculated that due to the decrease in intracellular glucose levels, the glycolytic pathway is inhibited. To generate sufficient glucose for maintaining normal physiological activities, the gluconeogenesis pathway is up-regulated.

To validate this hypothesis, we expanded the range of detected genes and selected a total of 15 genes involved in the insulin downstream phosphoinositide 3-kinase/protein kinase B (PI3K/Akt) signaling pathway, glycolysis, gluconeogenesis, glucose transport, glycogen synthesis, and glycogenolysis. The expression changes of these genes after long-term interference was measured. As expected, after *LvRTK2* interference, the expression of key genes in the insulin downstream signaling pathway, including PI3K, PDK1, and AKT, was significantly down-regulated in the experimental group. As a conserved key regulatory factor in the intracellular insulin signaling cascade, the down-regulation of AKT is believed to reduce the expression of the glucose transporters (GLUTs) [75]. This was confirmed in our results, as both AKT and GLUT expression were significantly down-regulated in the interference group. On the other hand, the down-regulation of AKT leads to an up-regulation of forkhead box transcription factor (FOXO) expression, which was originally suppressed by phosphorylated AKT [76]. The up-regulation of FOXO further regulates downstream pathways such as glycolysis and gluconeogenesis [77]. Specifically, our results showed that the expression of key genes in the glycolysis pathway, such as HK, Glucose-6-phosphate isomerase (G6PI), Phosphoglycerate kinase (PGK), and Pyruvate kinase (PK), was down-regulated after interference, and these genes are considered to encode crucial rate-limiting enzymes in the glycolysis process [74]. In addition, the expression levels of key genes in the gluconeogenesis pathway, such as Glucose-6-phosphatase (G6PC), Fructose 1, 6-bisphosphatase (FBP), PEPCK, and Pyruvate carboxylase (PC), were significantly up-regulated. Furthermore, the down-regulation of AKT expression was proved to inhibit the cAMP-response element binding protein (CREB) and activate glycogen synthase kinase (GSK), thereby inhibiting glycogen synthesis and energy storage [78,79]. This was also confirmed in our results, with the expression measurements of key genes in glycogen synthesis/glycogenolysis showing that the gene involved in glycogen synthesis, glycogen synthase (GYS), was significantly down-regulated, while the gene responsible for glycogenolysis, GSK, was activated.

All these results confirm our previous hypothesis that *LvRTK2* interference inhibits the insulin downstream PI3K/Akt signaling pathway, weakening the ability of glucose to enter the cell via GLUT, thereby reducing the intracellular glucose content. The decrease in intracellular glucose levels forces the inhibition of the glycolysis and glycogen synthesis process to avoid further glucose consumption. On the other hand, activation of the gluconeogenesis and glycogenolysis process enables shrimp to convert stored nutrients such as glycogen, fatty acids, and amino acids from the hepatopancreas and muscle into glucose to maintain normal physiological activities [80]. This metabolic disorder is considered the primary reason for the slow growth of shrimp caused by *LvRTK2* interference, indicating the crucial role of the insulin-like receptor in regulating shrimp growth.

In summary, long-term interference experiments indicate that the interference of *LvRTK2* significantly reduced the growth rate of shrimp. Meanwhile, short-term interference experiments suggest that *LvRTK2* is involved in the clearance of exogenous glucose in shrimp. Additionally, the dsLvRTK2 group exhibited lower glucose levels in the hepatopancreas, indicating that *LvRTK2* interference affects glucose uptake and utilization in shrimp. Further investigation of the expression of key enzymes and downstream genes related to glucose metabolism will help elucidate the potential mechanisms underlying the slow growth of shrimp. By analyzing these results (Section 3.8, Section 3.9, Section 3.10 and Section 3.11), we have not only established a connection between *LvRTK2* and shrimp growth but also gained a preliminary understanding of the signaling pathway through which *LvRTK2* is involved in the insulin regulation of *L. vannamei* growth. Based on these clues, we believe that *LvRTK2* can regulate the conserved insulin PI3K/Akt signaling cascade similar to that found in mammals and insects [9], and regulates shrimp growth through downstream molecular events, such as glycolysis, gluconeogenesis, glycogen synthesis, and glycogenolysis [74]. Accordingly, we created a schematic diagram illustrating the signaling pathway of shrimp growth and glucose metabolism regulated by insulin-like receptors (Figure 12).

## 5. Conclusions

This study first confirmed the existence of a second type of RTK insulin-like receptor in the decapod crustacean *L. vannamei*, with its sequence, structure, and function being highly conserved. Notably, this is the first characterization of RTK2 function in crustaceans, filling the research gap of IRs in crustacean glucose metabolism and growth regulation through in vivo and in vitro experiments. The significant growth inhibitory phenotype caused by *LvRTK2* silencing suggests that it may affect the normal growth of *L. vannamei*, indicating that RTKs are important growth regulatory factors in crustaceans. Further studies found that *LvRTK2* can regulate a series of glucose metabolism processes in shrimp through the conserved insulin PI3K/AKT signaling pathway, thereby controlling the growth of shrimp. The results reported in this study provide valuable information for understanding the growth regulatory mechanisms through the insulin signaling pathway in decapod crustaceans.

## Figures and Tables

**Table 1:** Information of primer sequences used in the present study. The forward and reverse primers are represented by F and R, respectively. The PCR product sizes, annealing temperatures (Tm), and the NCBI accession numbers of each gene are provided.

Primer Name	Primer Sequence (5′-3′)	Product Size (bp)	Tm (°C)	Accession No.
LvRTK2-F	TGTGTTGTGTTTATGTTCCTTGT	6439	48	PP932464.1
LvRTK2-R	CCATCTGAAAGGCACGTACATT			
Quantitative primers				
QLvRTK2-F	CCATCCACCCAGAGAAAC	179	52	PP932464.1
QLvRTK2-R	ACGGCTCGGACACTAAAG			
QLv18S-F	TATACGCTAGTGGAGCTGGAA	136	54	AF186250.1
QLv18S-R	GGGGAGGTAGTGACGAAAAAT			
QLvAKT-F	TCAGAATGTCCAAATCCAGCC	159	55	XM_027364781.1
QLvAKT-R	CCAAATGTCCCTTTCCCAAGT			
QLvFBP-F	AAGGAGAGGAGGTGAAGAAGC	202	55	XM_027380590.1
QLvFBP-R	CCTATGGAGACGAGGCAATC			
QLvFOXO-F	CCCGATACAGGACACGAT	203	51	XM_027372265.1
QLvFOXO-R	AAGTTGGGGTCAATCTCG			
QLvG6PC-F	AAAGTTGGAACCTGCGGA	255	54	XM_027351517.1
QLvG6PC-R	TCTCTCCCGTCCACCAAT			
QLvG6PI-F	GAGTTCTGGGACTGGGTT	169	48	XM_027372159.1
QLvG6PI-R	CAAGCAATACAGGAATGTTC			
QLvGLUT-F	ATTGTGACCCTCTATGTGG	287	51	XM_027383722.1
QLvGLUT-R	ACTGACCCTGTACCCTTG			
QLvGSK-F	GTTTCGGTGTTGTGTTCCA	182	51	XM_027362481.1
QLvGSK-R	CACTTCTTCTTTCTTGTCTCCA			
QLvGYS-F	GCCTCCCTGAACCAGATGAA	107	56	XM_027374375.1
QLvGYS-R	ATTGTGTGTGGTGATTGGCG			
QLvHK-F	GCTCGGTTTCACTTTCTC	229	50	XM_027378214.1
QLvHK-R	AGCACATCGCTTGTCCAT			
QLvPC-F	ATTGCTCAGTCCAGCGAAG	252	55	XM_027368506.1
QLvPC-R	GAGACTGTACCAAGTCAATCCC			
QLvPDK1-F	GCCAGCATCTGACCTCTG	228	48	XM_027383801.1
QLvPDK1-R	ATACTTGGATAGCCATTTCG			
QLvPEPCK-F	TGTCCAACACCATCTTCACC	138	54	XM_027371589.1
QLvPEPCK-R	TGCCCGATTCTTTGCTCC			
QLvPGK-F	CTTTCCTCCCTGACTGCG	112	56	XM_027383801.1
QLvPGK-R	TTGCCCTCCTCCTCTACG			
QLvPI3K-F	TGGGGAGAAAGAGTACCAG	201	50	XM_027362260.1
QLvPI3K-R	CCAATACCATTCCGCATC			
QLvPK-F	GAAGCCTCATTCAAAGCA	190	49	XM_027381851.1
QLvPK-R	CAGCATTCTGAGGCACAG			
RNAi primers				
dsLvRTK2-F	TAATACGACTCACTATAGGGGAGGGTCGTCTCATCATC	677	60	PP932464.1
dsLvRTK2-R	TAATACGACTCACTATAGGGTAAAAGGCGTATTTCGTG			

## Data Availability

Data is contained within the article.

## Supplementary Materials

The following supporting information can be downloaded at: https://www.mdpi.com/article/10.3390/biom14101300/s1, Figure S1: Multiple sequence alignment of predicted phosphorylation sites and glycosylation sites of decapoda RTK2. The predicted phosphorylated tyrosine residues in the intracellular region are indicated by blue boxes, and the glycosylated asparagine residues predicted in the extracellular region are indicated by red boxes.

## References

[1] Saltiel A.R. (2016). Insulin signaling in the control of glucose and lipid homeostasis. Handbook of Experimental Pharmacology.

[2] Vecchio I., Tornali C., Bragazzi N.L., Martini M. (2018). The discovery of insulin: An important milestone in the history of medicine. Front. Endocrinol..

[3] Wilson L.M., Castle J.R. (2020). Recent advances in insulin therapy. Diabetes Technol. Ther..

[4] Doležel D., Hanus R., Fiala I., Beneš V., Vaněčková H., Horák A., Lukšan O., Jedlička P., Bazalová O., Provazník J. (2020). Complex evolution of insect insulin receptors and homologous decoy receptors, and functional significance of their multiplicity. Mol. Biol. Evol..

[5] Bedinger D.H., Adams S.H. (2015). Metabolic, anabolic, and mitogenic insulin responses: A tissue-specific perspective for insulin receptor activators. Mol. Cell. Endocrinol..

[6] LeRoith D., Holly J.M.P., Forbes B.E. (2021). Insulin-like growth factors: Ligands, binding proteins, and receptors. Mol. Metab..

[7] Sang M., Li C., Wu W., Li B. (2016). Identification and evolution of two insulin receptor genes involved in *Tribolium castaneum* development and reproduction. Gene.

[8] Claeys I., Simonet G., Poels J., Van Loy T., Vercammen L., De Loof A., Vanden Broeck J. (2002). Insulin-related peptides and their conserved signal transduction pathway. Peptides.

[9] Brown M.R., Clark K.D., Gulia M., Zhao Z., Garczynski S.F., Crim J.W., Suderman R.J., Strand M.R. (2008). An insulin-like peptide regulates egg maturation and metabolism in the mosquito aedes aegypti. Proc. Natl. Acad. Sci. USA.

[10] Veenstra J.A. (2020). Arthropod igf, relaxin and gonadulin, putative orthologs of drosophila insulin-like peptides 6, 7 and 8, likely originated from an ancient gene triplication. PeerJ.

[11] Haeusler R.A., McGraw T.E., Accili D. (2018). Biochemical and cellular properties of insulin receptor signalling. Nat. Rev. Mol. Cell Biol..

[12] Okuyama T., Kyohara M., Terauchi Y., Shirakawa J. (2021). The roles of the igf axis in the regulation of the metabolism: Interaction and difference between insulin receptor signaling and igf-i receptor signaling. Int. J. Mol. Sci..

[13] Makhijani P., Basso P.J., Chan Y.T., Chen N., Baechle J., Khan S., Furman D., Tsai S., Winer D.A. (2023). Regulation of the immune system by the insulin receptor in health and disease. Front. Endocrinol..

[14] Iizuka Y., Ueda Y., Yagi Y., Sakurai E. (2010). Significant improvement of insulin resistance of gk rats by treatment with sodium selenate. Biol. Trace Elem. Res..

[15] Zhang X., Zhu X., Bi X., Huang J., Zhou L. (2022). The insulin receptor: An important target for the development of novel medicines and pesticides. Int. J. Mol. Sci..

[16] Pollak M. (2012). The insulin and insulin-like growth factor receptor family in neoplasia: An update. Nat. Rev. Cancer.

[17] Erion R., Sehgal A. (2013). Regulation of insect behavior via the insulin-signaling pathway. Front. Physiol..

[18] Zhao H., Chen L., Liu M., Zhao S., Ma W., Jiang Y. (2024). Insulin receptor participates in the peripheral olfactory processes of honey bees (*Apis cerana cerana*). Insect Sci..

[19] Silva-Oliveira G., De Paula I.F., Medina J.M., Alves-Bezerra M., Gondim K.C. (2021). Insulin receptor deficiency reduces lipid synthesis and reproductive function in the insect *Rhodnius prolixus*. Biochim. Biophys. Acta. Mol. Cell Biol. Lipids.

[20] Veenstra J.A. (2020). Gonadulins, the fourth type of insulin-related peptides in decapods. Gen. Comp. Endocrinol..

[21] Guo Q., Li S., Lv X., Xiang J., Sagi A., Manor R., Li F. (2018). A putative insulin-like androgenic gland hormone receptor gene specifically expressed in male Chinese shrimp. Endocrinology.

[22] Aizen J., Chandler J.C., Fitzgibbon Q.P., Sagi A., Battaglene S.C., Elizur A., Ventura T. (2016). Production of recombinant insulin-like androgenic gland hormones from three decapod species: In vitro testicular phosphorylation and activation of a newly identified tyrosine kinase receptor from the eastern spiny lobster, *Sagmariasus verreauxi*. Gen. Comp. Endocrinol..

[23] Chen Y.-L., Wang Y.-M., Xu H.-J., Li J.-W., Luo J.-Y., Wang M.-R., Ma W.-M. (2022). The characterization and knockdown of a male gonad-specific insulin-like receptor gene in the white shrimp *Penaeus vannamei*. Aquac. Rep..

[24] Liu A., Hao S., Liu F., Huang H., Ye H. (2023). Isolation of an insulin-like receptor involved in the testicular development of the mud crab *Scylla paramamosain*. Int. J. Mol. Sci..

[25] Manor R., Weil S., Oren S., Glazer L., Aflalo E.D., Ventura T., Chalifa-Caspi V., Lapidot M., Sagi A. (2007). Insulin and gender: An insulin-like gene expressed exclusively in the androgenic gland of the male crayfish. Gen. Comp. Endocrinol..

[26] Ventura T., Manor R., Aflalo E.D., Weil S., Rosen O., Sagi A. (2012). Timing sexual differentiation: Full functional sex reversal achieved through silencing of a single insulin-like gene in the prawn, *Macrobrachium rosenbergii*. Biol. Reprod..

[27] Sharabi O., Manor R., Weil S., Aflalo E.D., Lezer Y., Levy T., Aizen J., Ventura T., Mather P.B., Khalaila I. (2016). Identification and characterization of an insulin-like receptor involved in crustacean reproduction. Endocrinology.

[28] Li J., Tian Y., Li X., Zuo J., Zhao R., Sun J. (2022). Insulin-like signaling promotes limb regeneration in the Chinese mitten crab (*Eriocheir sinensis*). Fish Shellfish. Immunol..

[29] Xu H.-J., Xue J., Lu B., Zhang X.-C., Zhuo J.-C., He S.-F., Ma X.-F., Jiang Y.-Q., Fan H.-W., Xu J.-Y. (2015). Two insulin receptors determine alternative wing morphs in planthoppers. Nature.

[30] Nässel D.R., Vanden Broeck J. (2016). Insulin/igf signaling in drosophila and other insects: Factors that regulate production, release and post-release action of the insulin-like peptides. Cell. Mol. Life Sci. CMLS.

[31] Xu H.J., Zhang C.X. (2017). Insulin receptors and wing dimorphism in rice planthoppers. Philos. Trans. R. Soc. B Biol. Sci..

[32] Lu H.-L., Pietrantonio P.V. (2011). Insect insulin receptors: Insights from sequence and caste expression analyses of two cloned hymenopteran insulin receptor cdnas from the fire ant. Insect Mol. Biol..

[33] Jedlička P., Ernst U.R., Votavová A., Hanus R., Valterová I. (2016). Gene expression dynamics in major endocrine regulatory pathways along the transition from solitary to social life in a bumblebee, *Bombus terrestris*. Front. Physiol..

[34] Jung H., Lyons R.E., Hurwood D.A., Mather P.B. (2013). Genes and growth performance in crustacean species: A review of relevant genomic studies in crustaceans and other taxa. Rev. Aquac..

[35] Amente L.D., Mills N.T., Le T.D., Hyppönen E., Lee S.H. (2024). Unraveling phenotypic variance in metabolic syndrome through multi-omics. Hum. Genet..

[36] Zhang X., Yuan J., Sun Y., Li S., Gao Y., Yu Y., Liu C., Wang Q., Lv X., Zhang X. (2019). Penaeid shrimp genome provides insights into benthic adaptation and frequent molting. Nat. Commun..

[37] Livak K.J., Schmittgen T.D. (2001). Analysis of relative gene expression data using real-time quantitative pcr and the 2(-delta delta c(t)) method. Methods.

[38] Gao Y., Wei J., Yuan J., Zhang X., Li F., Xiang J. (2017). Transcriptome analysis on the exoskeleton formation in early developmetal stages and reconstruction scenario in growth-moulting in *Litopenaeus vannamei*. Sci. Rep..

[39] Brown J., Pirrung M., McCue L.A. (2017). Fqc dashboard: Integrates fastqc results into a web-based, interactive, and extensible fastq quality control tool. Bioinformatics.

[40] Langdon W.B. (2015). Performance of genetic programming optimised bowtie2 on genome comparison and analytic testing (gcat) benchmarks. Biodata Min..

[41] Kim D., Paggi J.M., Park C., Bennett C., Salzberg S.L. (2019). Graph-based genome alignment and genotyping with hisat2 and hisat-genotype. Nat. Biotechnol..

[42] Pertea M., Kim D., Pertea G.M., Leek J.T., Salzberg S.L. (2016). Transcript-level expression analysis of rna-seq experiments with hisat, stringtie and ballgown. Nat. Protoc..

[43] Duan H., Jin S., Li F., Zhang X., Xiang J. (2018). Neuroanatomy and morphological diversity of brain cells from adult crayfish *Cherax quadricarinatus*. J. Oceanol. Limnol..

[44] Chen T., Wong N.-K., Jiang X., Luo X., Zhang L., Yang D., Ren C., Hu C. (2015). Nitric oxide as an antimicrobial molecule against vibrio harveyi infection in the hepatopancreas of pacific white shrimp, *Litopenaeus vannamei*. Fish Shellfish Immunol..

[45] Guo Q., Li S., Lv X., Xiang J., Manor R., Sagi A., Li F. (2019). Sex-biased chhs and their putative receptor regulate the expression of iag gene in the shrimp *Litopenaeus vannamei*. Front. Physiol..

[46] Gutiérrez A., Nieto J., Pozo F., Stern S., Schoofs L. (2007). Effect of insulin/igf-i like peptides on glucose metabolism in the white shrimp *Penaeus vannamei*. Gen. Comp. Endocrinol..

[47] Pan X., Pei Y., Zhang C., Huang Y., Chen L., Wei L., Li C., Dong X., Chen X. (2022). Effect of insulin receptor on juvenile hormone signal and fecundity in *Spodoptera litura* (f.). Insects.

[48] Vitali V., Horn F., Catania F. (2018). Insulin-like signaling within and beyond metazoans. Biol. Chem..

[49] Veenstra J.A. (2016). Similarities between decapod and insect neuropeptidomes. PeerJ.

[50] Veenstra J.A. (2015). The power of next-generation sequencing as illustrated by the neuropeptidome of the crayfish *Procambarus clarkii*. Gen. Comp. Endocrinol..

[51] Chandler J.C., Aizen J., Elizur A., Hollander-Cohen L., Battaglene S.C., Ventura T. (2015). Discovery of a novel insulin-like peptide and insulin binding proteins in the eastern rock lobster *Sagmariasus verreauxi*. Gen. Comp. Endocrinol..

[52] Gao Y., Zhang X., Yuan J., Zhang C., Li S., Li F. (2022). Crispr/cas9-mediated mutation on an insulin-like peptide encoding gene affects the growth of the ridgetail white prawn *Exopalaemon carinicauda*. Front. Endocrinol..

[53] Ou J., Deng H.-M., Zheng S.-C., Huang L.-H., Feng Q.-L., Liu L. (2014). Transcriptomic analysis of developmental features of bombyx mori wing disc during metamorphosis. BMC Genom..

[54] Wertheimer E., Trebicz M., Eldar T., Gartsbein M., Nofeh-Moses S., Tennenbaum T. (2000). Differential roles of insulin receptor and insulin-like growth factor-1 receptor in differentiation of murine skin keratinocytes. J. Investig. Dermatol..

[55] Kimura K.D., Tissenbaum H.A., Liu Y., Ruvkun G. (1997). Daf-2, an insulin receptor-like gene that regulates longevity and diapause in *Caenorhabditis elegans*. Science.

[56] Xu H.-J., Li J.-W., Chen Y.-L., Yang J.-S., Ma W.-M., Qian G.-Y. (2021). A novel uniquely ovary-expressed insulin-like receptor in the female prawn, *Macrobrachium rosenbergii* (Decapoda, Palaemonidae). Crustaceana.

[57] McKern N.M., Lawrence M.C., Streltsov V.A., Lou M.Z., Adams T.E., Lovrecz G.O., Elleman T.C., Richards K.M., Bentley J.D., Pilling P.A. (2006). Structure of the insulin receptor ectodomain reveals a folded-over conformation. Nature.

[58] Gao Y., Zhang X., Wei J., Sun X., Yuan J., Li F., Xiang J. (2015). Whole transcriptome analysis provides insights into molecular mechanisms for molting in *Litopenaeus vannamei*. PLoS ONE.

[59] Xu K., Morgan K.T., Todd Gehris A., Elston T.C., Gomez S.M. (2011). A whole-body model for glycogen regulation reveals a critical role for substrate cycling in maintaining blood glucose homeostasis. PLoS Comput. Biol..

[60] Ye J., Medzhitov R. (2019). Control strategies in systemic metabolism. Nat. Metab..

[61] Kim Y., Hong Y. (2015). Regulation of hemolymph trehalose level by an insulin-like peptide through diel feeding rhythm of the beet armyworm, *Spodoptera exigua*. Peptides.

[62] Gallardo N., Carrillo O., Moltó E., Deás M., González-Suárez R., Carrascosa J.M., Ros M., Andrés A. (2003). Isolation and biological characterization of a 6-kda protein from hepatopancreas of lobster *Panulirus argus* with insulin-like effects. Gen. Comp. Endocrinol..

[63] Su M., Zhang X., Yuan J., Zhang X., Li F. (2022). The role of insulin-like peptide in maintaining hemolymph glucose homeostasis in the pacific white shrimp *Litopenaeus vannamei*. Int. J. Mol. Sci..

[64] Haselton A.T., Fridell Y.-W.C. (2011). Insulin injection and hemolymph extraction to measure insulin sensitivity in adult drosophila melanogaster. J. Vis. Exp..

[65] Chung J.S. (2014). An insulin-like growth factor found in hepatopancreas implicates carbohydrate metabolism of the blue crab *Callinectes sapidus*. Gen. Comp. Endocrinol..

[66] Trapp M., Valle S.C., Poppl A.G., Chitto A.L.F., Kucharski L.C., Da Silva R.S.M. (2018). Insulin-like receptors and carbohydrate metabolism in gills of the euryhaline crab *Neohelice granulata*: Effects of osmotic stress. Gen. Comp. Endocrinol..

[67] Friedman M.I., Ramirez I., Wade G.N., Siegel L.I., Granneman J. (1982). Metabolic and physiologic effects of a hunger-inducing injection of insulin. Physiol. Behav..

[68] Han H.-S., Kang G., Kim J.S., Choi B.H., Koo S.-H. (2016). Regulation of glucose metabolism from a liver-centric perspective. Exp. Mol. Med..

[69] Jiang Q., Ji P., Ao S., Gao X., Zhang X. (2023). Effects of starvation and refeeding on glucose metabolism and immune responses in *Macrobrachium rosenbergii*. Mar. Biotechnol..

[70] Su M., Zhang X., Zhang X., Yuan J., Yang M., Li F. (2024). Comparative transcriptome analysis provides a glance into the regulatory of insulin-like peptide 1 gene in the pacific white shrimp *Litopenaeus vannamei*. Aquaculture.

[71] Wang T., Yu Y., Li S.H., Li F.H. (2024). Molecular mechanisms of sex determination and differentiation in decapod crustaceans for potential aquaculture applications: An overview. Rev. Aquac..

[72] Flores K.A., Pérez-Moreno J.L., Durica D.S., Mykles D.L. (2024). Phylogenetic and transcriptomic characterization of insulin and growth factor receptor tyrosine kinases in crustaceans. Front. Endocrinol..

[73] Hammond S.M., Bernstein E., Beach D., Hannon G.J. (2000). An RNA-directed nuclease mediates post-transcriptional gene silencing in *Drosophila* cells. Nature.

[74] Lu J., Tao X., Li M., Zhang X., Zhou Q., Luo J., Zhu T., Jiao L., Jin M. (2023). Dietary inositol improved glucose and lipid metabolism mediated by the insulin-pi3k-akt signaling pathway in pacific white shrimp (*Litopenaeus vannamei*). Aquaculture.

[75] Wang W., Shi B., Cong R., Hao M., Peng Y., Yang H., Song J., Feng D., Zhang N., Li D. (2022). Ring-finger e3 ligases regulatory network in pi3k/akt-mediated glucose metabolism. Cell Death Discov..

[76] Nijhout H.F., Callier V. (2013). A new mathematical approach for qualitative modeling of the insulin-tor-mapk network. Front. Physiol..

[77] Titchenell P.M., Lazar M.A., Birnbaum M.J. (2017). Unraveling the regulation of hepatic metabolism by insulin. Trends Endocrinol. Metab..

[78] Defferrari M.S., Da Silva S.R., Orchard I., Lange A.B. (2018). A *Rhodnius prolixus* insulin receptor and its conserved intracellular signaling pathway and regulation of metabolism. Front. Endocrinol..

[79] Lin J.-L., Lin P.-L., Gu S.-H. (2009). Phosphorylation of glycogen synthase kinase-3β in relation to diapause processing in the silkworm, bombyx mori. J. Insect Physiol..

[80] Hernández-Aguirre L.E., Cota-Ruiz K., Peregrino-Uriarte A.B., Gómez-Jiménez S., Yepiz-Plascencia G. (2021). The gluconeogenic glucose-6-phosphatase gene is expressed during oxygen-limited conditions in the white shrimp *Penaeus* (*Litopenaeus*) *vannamei*: Molecular cloning, membrane protein modeling and transcript modulation in gills and hepatopancreas. J. Bioenerg. Biomembr..

