# Supplementary material for: Identification and Characterization of a Novel Insulin-like Receptor (LvRTK2) Involved in Regulating Growth and Glucose Metabolism of the Pacific White Shrimp Litopenaeus vannamei
Source: Biomolecules. 2024 Oct 14;14(10):1300. doi: 10.3390/biom14101300 (PMC11506343; doi:10.3390/biom14101300)
Supplement: Supplementary file 1 [file biomolecules-14-01300-s001.zip › Supplementary figure.pdf]

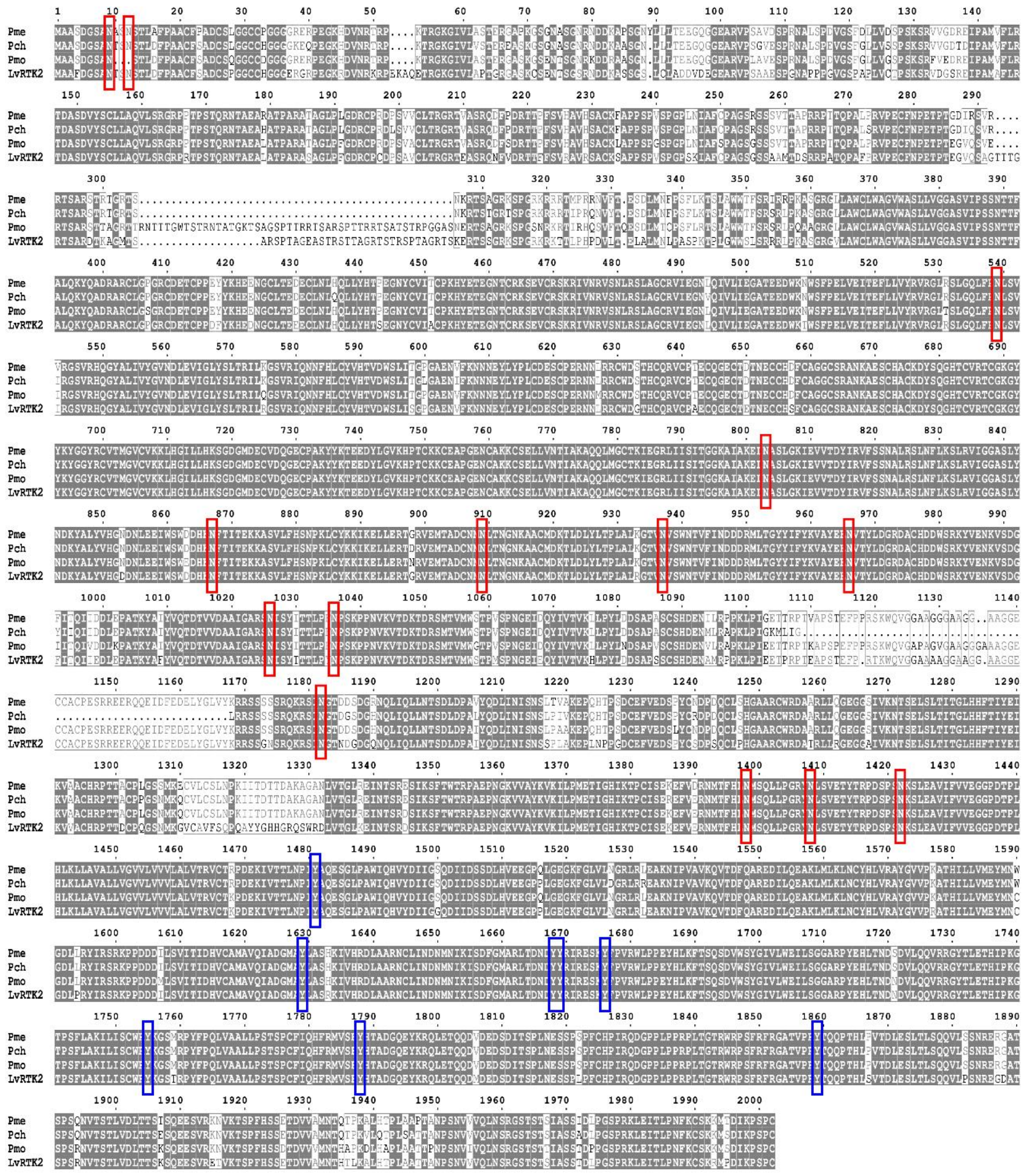

Figure S1. Multiple sequence alignment of predicted phosphorylation sites and glycosylation sites of decapoda RTK2. The predicted phosphorylated tyrosine residues in the intracellular region are indicated by blue boxes, and the glycosylated asparagine residues predicted in the extracellular region are indicated by red boxes.
